# Efficacy, safety and pharmacokinetics of Unecritinib (TQ-B3101) for patients with *ROS1* positive advanced non-small cell lung cancer: a Phase I/II Trial

**DOI:** 10.1038/s41392-023-01454-z

**Published:** 2023-06-30

**Authors:** Shun Lu, Hongming Pan, Lin Wu, Yu Yao, Jianxing He, Yan Wang, Xiuwen Wang, Yong Fang, Zhen Zhou, Xicheng Wang, Xiuyu Cai, Yan Yu, Zhiyong Ma, Xuhong Min, Zhixiong Yang, Lejie Cao, Huaping Yang, Yongqian Shu, Wu Zhuang, Shundong Cang, Jian Fang, Kai Li, Zhuang Yu, Jiuwei Cui, Yang Zhang, Man Li, Xinxuan Wen, Jie Zhang, Weidong Li, Jianhua Shi, Xingxiang Xu, Diansheng Zhong, Tao Wang, Jiajia Zhu

**Affiliations:** 1 Department of Medical Oncology, Shanghai Chest Hospital, School of Medicine, Shanghai Jiao Tong University, 200030 Shanghai, China; 2 Department of Medical Oncology, Sir Run Run Shaw Hospital, Zhejiang University, 310016 Hangzhou, China; 3 Department of Thoracic Medical Oncology, The Affiliated Cancer Hospital of Xiangya School of Medicine, Central South University (Hunan Cancer Hospital), 410031 Changsha, China; 4 Department of Medical Oncology, The First Affiliated Hospital of Xi’an Jiaotong University, 710061 Xian, China; 5 Department of Thoracic Surgery, The First Affiliated Hospital of Guangzhou Medical University, 510120 Guangzhou, China; 6 Department of Medical Oncology, National Cancer Center/National Clinical Research Center for Cancer/Cancer Hospital, Chinese Academy of Medical Sciences and Peking Union Medical College, 100021 Beijing, China; 7 Department of Oncology, Qilu Hospital of Shandong University, 250012 Jinan, China; 8 Department of Oncology, The First Affiliated Hospital/School of Clinical Medicine of Guangdong Pharmaceutical University, 510699 Guangzhou, China; 9 Department of Medical Oncology, Sun Yat-sen University Cancer Center, 510060 Guangzhou, China; 10 Department of Respiratory Medicine, Harbin Medical University Cancer Hospital, 150081 Harbin, China; 11 Department of Medical Oncology, Henan Tumor Hospital, 450003 Zhengzhou, China; 12 Department of Oncology Radiotherapy, Anhui Chest Hospital, 230022 Hefei, China; 13 Department of Cancer Center, Affiliated Hospital of Guangdong Medical University, 524000 Zhanjiang, China; 14 Department of Respiratory Medicine, The First Affiliated Hospital of the University of Science and Technology of China, Anhui Provincial Hospital, 230031 Hefei, China; 15 Department of Respiratory and Critical Care Medicine, Xiangya Hospital Central South University, 410008 Changsha, China; 16 Department of Cancer Center, Jiangsu Province Hospital, 210029 Nanjing, China; 17 Department of Thoracic Oncology, Fujian Cancer Hospital and Fujian Medical University Cancer Hospital, 350014 Fuzhou, China; 18 Department of Medical Oncology, Henan Province People’s Hospital, 450003 Zhengzhou, China; 19 Department of Thoracic Oncology, Peking University Cancer Hospital, 100142 Beijing, China; 20 Department of Pulmonary Oncology, Tianjin Medical University Cancer Institute and Hospital, 300060 Tianjin, China; 21 Department of Oncology, The Affiliated Hospital of Qingdao University, 266000 Qingdao, China; 22 Department of Oncology, The First Hospital of Jilin University, 130061 Changchun, China; 23 Department of Medical Oncology, The Second Hospital of Dalian Medical University, 116023 Dalian, China; 24 Department of Oncology, Xiangyang No. 1 People’s Hospital, 441011 Xiangyang, China; 25 Department of Respiratory and Critical Care Medicine, The Second Hospital of Jilin University, 130041 Changchun, China; 26 Department of Medical Oncology, Affiliated Cancer Hospital and Institute of Guangdong Medical University, 510095 Guangzhou, China; 27 Department of Oncology, Linyi Cancer Hospital, 276002 Linyi, China; 28 Department of Respiratory and Critical Care Medicine, Northern Jiangsu People’s Hospital, 225001 Yangzhou, China; 29 Department of Medical Oncology, Tianjin Medical University General Hospital, 300052 Tianjin, China; 30 Biostatistics Department of Clinical Center of Research Institute, Chia Tai Tianqing Pharmaceutical Group Co., Ltd., 222000 Nanjing, China

**Keywords:** Lung cancer, Drug development

## Abstract

This phase I/II trial characterized the tolerability, safety, and antitumor activities of unecritinib, a novel derivative of crizotinib and a multi-tyrosine kinase inhibitor targeting ROS1, ALK, and c-MET, in advanced tumors and ROS1 inhibitor-naive advanced or metastatic non-small cell lung cancer (NSCLC) harboring *ROS1* rearrangements. Eligible patients received unecritinib 100, 200, and 300 mg QD, and 200, 250, 300, and 350 mg BID in a 3 + 3 design during dose escalation and 300 and 350 mg BID during expansion. Phase II trial patients received unecritinib 300 mg BID in continuous 28-day cycles until disease progression or unacceptable toxicity. The primary endpoint was the objective response rate (ORR) per independent review committee (IRC). Key secondary endpoints included intracranial ORR and safety. The ORR of 36 efficacy evaluable patients in the phase I trial was 63.9% (95% CI 46.2%, 79.2%). In the phase II trial, 111 eligible patients in the main study cohort received unecritinib. The ORR per IRC was 80.2% (95% CI 71.5%, 87.1%) and the median progression-free survival (PFS) per IRC was 16.5 months (95% CI 10.2, 27.0). Additionally, 46.9% of the patients who received recommended phase II dose of 300 mg BID experienced grade 3 or higher treatment-related adverse events. Treatment-related ocular disorders and neurotoxicity occurred in 28.1% and 34.4% of patients, respectively, but none was grade 3 or higher. Unecritinib is efficacious and safe for ROS1 inhibitor-naive patients with *ROS1*-positive advanced NSCLC, particularly patients with brain metastases at baseline, strongly supporting that unecritinib should become one of the standards of care for *ROS1*-positive NSCLC.

ClinicalTrials.gov identifier: NCT03019276 and NCT03972189.

## Introduciton

Lung cancer remains the leading cause of cancer death globally, accounting for approximately one in five (18.0%) of the total cancer deaths,^1^ and is expected to be the principal cause of death in both sexes in China and the USA in 2022.^2^ Recent remarkable advances in molecular targeted therapies have significantly extended the survival of patients with lung cancer harboring actionable mutations including *EGFR* exon 19 deletion and L858R mutations, and *ALK* and *ROS1* rearrangements.^3–7^ The *ROS1* oncogene, which is located on chromosome 6 (6q22), encodes protooncogene receptor tyrosine kinase ROS1. The kinase is composed of an intracellular *C*-terminus containing the kinase domain, a single transmembrane domain, and an extracellular *N*-terminal domain^8^ and is constitutively activated by fusion partner proteins as a result of chromosomal rearrangements that occur in approximately 1% to 2% of patients with non-small cell lung cancer (NSCLC).^8–10^ Among diverse ROS1 fusions, CD74 is the most frequent ROS1 fusion partner, and *CD74–ROS1* fusion is present in ~44% of NSCLC cases with *ROS1* rearrangements.^9^ Intriguingly, we have previously noted that NSCLC patients carrying *CD74–ROS1* fusion are more likely to develop brain metastases.^11^ Targeted therapies are well established for NSCLC with *ALK* and *ROS1* rearrangements that lead to constitutive kinase activity of ALK or ROS1.^6^ Crizotinib, an ALK and ROS1 tyrosine kinase inhibitor (TKI), has become not only one of the standards of care for *ALK-*positive but also the preferred drug for *ROS1*-rearranged NSCLC patients.^12^

Several pivotal trials, including the METROS trial,^13^ the PROFILE 1001 study^14^ and the OO 12-01 study,^15^ have demonstrated that crizotinib elicits a durable response and extends the progression-free survival (PFS) of patients with *ROS1*-rearranged advanced NSCLC, establishing ROS1 as a valid therapeutic target in *ROS1*-rearranged lung cancers. However, despite significant improvement in the objective response rate (ORR) and survival outcome of *ROS1*-rearranged NSCLC patients, there are concerning treatment-related adverse events (TRAEs) such as prominent gastrointestinal disturbances and ocular toxicities with crizotinib^12,16^ and neurotoxicity with entrectinib.^17^

Unecritinib (TQ-B3101), a multi-TKI targeting ROS1, ALK, and c-MET, is a derivative of crizotinib *via* structural modification of the pyridine ring. Our in vitro study showed that similar to crizotinib, unecritinib inhibited AKT phosphorylation and its downstream signaling molecules ERK1/2 (extracellular signal-regulated protein kinases 1 and 2) (Supplementary Fig. S1). Furthermore, unecritinib had an IC_50_ of 142.7 nM and an IC_90_ of 821.3 nM while unecritinib M had an IC_50_ of 0.8 nM and an IC_90_ of 6.2 nM for wildtype ROS1. Unecritinib also displayed notable growth inhibitory effects on several cancer cell lines carrying *ALK* rearrangements or mutations or overexpressing c-MET, with an IC_50_ of 180 to 378.9 nm for lung cancer cells and an IC_50_ of 23.5 nm for gastric cancer cells overexpressing c-MET. The IC_50_ of growth inhibition by unecritinib is comparable to that by crizotinib (Supplementary Table S1).

Meanwhile, our preclinical studies showed a greater bioavailability of unecritinib than crizotinib in beagles (data not published). In mouse xenografts bearing human lung adenocarcinoma NCI-H3122 cells, unecritinib showed greater tumor growth inhibition than crizotinib, which may be explained by higher tumor tissue exposure to unecritinib *vs.* crizotinib (data not published). Notably, unecritinib M, the metabolite of unecritinib, had a considerably lower AUC_ocular tissue_/AUC_plasma_ than crizotinib in rats (data not published). The remarkably smaller distribution of unecritinib M in the ocular tissues suggests a lower risk of ocular toxicities. Indeed, no apparent ocular toxicities were observed in rats and beagles receiving unecritinib for 4 weeks (rats 135 mg/kg; beagles 81 mg/kg) (data not published).

In this phase I/II study, we aimed to characterize the tolerability, safety, and antitumor activities of unecritinib in ROS1 inhibitor-naive patients with *ROS1*-rearranged advanced NSCLC and other selected advanced solid tumors who had failed standard chemotherapy. As *CD74* is the most frequent *ROS1* fusion partner and *CD74*-*ROS1* fusion is present in ~44% of NSCLC cases with *ROS1* rearrangements,^18,19^ we also carried out a prespecified analysis of the efficacy of unecritinib for NSCLC cases with *CD74-ROS1* fusion.

## Results

### Patient characteristics

The phase I/II trial was conducted between July 12, 2017, and March 2, 2021, and the cutoff date for this analysis was December 20, 2021. Patient demographic, baseline, and prior treatment characteristics are described in Table 1 and the study flowcharts are shown in Supplementary Fig. S2. Twenty patients were enrolled in the dose escalation portion and 19 patients in the dose expanse portion of the phase I trial. Two of them (5.1%) had stage III tumors and 37 (94.9%) had stage IV tumors, and 61.5% received prior chemotherapy.

**Table 1:** Baseline patient characteristics

Characteristic	Phase I			Phase II
	Escalation phase	Expansion phase	All patients	
No. of patients	20	19	39	111
*Age, years*				
Median	48.0	58.0	52.0	52.0
Range	28–70	31–73	28–73	28–76
<65	17 (85.0)	13 (68.4)	30 (76.9)	94 (84.7)
≥65	3 (15.0)	6 (31.6)	9 (23.1)	17 (15.3)
*Sex*				
Male	9 (45.0)	10 (52.6)	19 (48.7)	43 (38.7)
Female	11 (55.0)	9 (47.4)	20 (51.3)	68 (61.3)
*Ethnicities*				
Han Chinese	20 (100.0)	19 (100.0)	39 (100.0)	105 (94.6)
Others	0 (0.0)	0 (0.0)	0 (0.0)	6 (5.4)
*ECOG PS*				
0	2 (10.0)	0 (0.0)	2 (5.1)	32 (28.8)
1	18 (90.0)	19 (100.0)	37 (94.9)	79 (71.2)
*Smoking history*				
Never smokers	18 (90.0)	14 (73.7)	32 (82.1)	80 (72.1)
Ever smokers	1 (5.0)	5 (26.3)	6 (15.4)	28 (25.2)
Current smokers	1 (5.0)	0 (0.0)	1 (2.6)	3 (2.7)
*Histologic classification*				
Adenocarcinoma of the lungs	19 (95.0)	16 (84.2)	35 (89.7)	110 (99.1)
Squamous cell carcinoma of the lung	1 (5.0)	0 (0.0)	1 (2.6)	0 (0.0)
Others	0 (0.0)	3 (15.8)^a^	3 (7.7)	1 (0.90)
*Stage of disease at trial entry*				
III	1 (5.0)	1 (5.3)	2 (5.1)	8 (7.2)
IV	19 (95.0)	18 (94.7)	37 (94.9)	103 (92.8)
Brain metastases at baseline	6 (30.0)	3 (15.8)	9 (23.1)	33 (29.7)
Liver metastases at baseline	–	–	–	15 (13.5)
*Prior treatments*				
Surgery	12 (60.0)	8 (42.1)	20 (51.3)	28 (25.2)
Chemotherapy	14 (70.0)	10 (52.6)	24 (61.5)	48 (43.2)
Radiotherapy	4 (20.0)	3 (15.8)	7 (17.9)	9 (8.1)
Other anti-tumor therapy^b^	6 (30.0)	4 (21.1)	10 (25.6)	28 (25.2)
*No. of prior regimens for advanced disease*				
0	NA	NA	NA	65 (58.6)
1	NA	NA	NA	35 (31.5)
2	NA	NA	NA	11 (9.9)
Gene fusions^c^				
*CD74-ROS1*	–	–	–	27 (67.5)
*SDC4-ROS1*	–	–	–	5 (12.5)
*SLC34A2-ROS1*	–	–	–	3 (7.5)
*EZR-ROS1*	–	–	–	1 (2.5)
*TPM3-ROS1*	–	–	–	1 (2.5)
*EZR-ROS1* and *TGFBR1-ROS1*^d^	–	–	–	1 (2.5)
*EZR-ROS1* and *SLC34A2-ROS1*	–	–	–	1 (2.5)
*TPM3/LRIG3/GOPC-ROS1*^e^	–	–	–	1 (2.5)

Eastern Cooperative Oncology Group (ECOG) performance-status (PS) scores range from 0 to 5, with higher numbers indicating increasing impairment in activities of daily living

*ECOG PS* Eastern Cooperative Oncology Group performance status, *NA* not available

^a^Including malignant melanoma (*n* = 1) and gastric adenocarcinoma (*n* = 2)

^b^Refers to EGFR-TKIs, immune checkpoint inhibitors, traditional Chinese medicine and antiangiogenic therapy

^c^40 patients with *ROS1* rearrangement detected by AmoyDx assay at the central laboratory subsequently underwent central testing by next-generation sequencing (NGS) and another 71 patients did not undergo NGS testing

^d^The patient carried both *EZR-ROS1* and *TGFBR1-ROS1*. The *EZR-ROS1* fusion affects the kinase domain while *TGFBR1-ROS1* fusion does not as fusion occurred at the 5’

^e^The fusion as detected by AmoyDx assay and the patient could carry any one of the fusions as the assay does not report these three fusions (*TPM3/LRIG3/GOPC*) individually

One hundred sixty patients were screened for eligibility for the phase II trial and 113 patients were eligible and received unecritinib, including 2 patients in the exploratory study cohort and 111 patients in the main study cohort. In the main study cohort, 103 patients (92.8%) had stage IV NSCLC. Thirty-three patients (29.7%) had brain metastasis and 15 (13.5%) had liver metastasis. Forty-eight patients (43.2%) received prior chemotherapy. Forty patients with *ROS1* rearrangement detected by AmoyDx assay at the central laboratory subsequently underwent central testing by NGS. The concordance rate was 100% between the Amoydx assay and central NGS testing (excluding samples that failed quality control). Twenty-seven patients (67.5%) had *CD74-ROS1* fusion. Five patients (12.5%) had *SDC4-ROS1* fusion, and 3 (7.5%) had *SLC34A2-ROS1* fusion. In addition, *EZR-ROS1* and *TPM3-ROS1* fusion was each present in 1 patient (2.5%). Furthermore, one patient carried both *EZR-ROS1* and *TGFBR1-ROS1* fusions and another carried both *EZR-ROS1* and *SLC34A2-ROS1* fusions. *ROS1* fusion partners were indeterminate in 71 patients who did not undergo NGS.

### Safety

The safety population was composed of 128 patients, including three patients who received unecritinib 300 mg BID during dose escalation, 12 patients who received unecritinib 300 mg BID during dose expansion, and 113 patients from the phase 2 trial. Data on gene mutations were available from six patients from the phase 1 trial, including 3 cases who had *ALK* fusion, 2 cases who had unspecified *ALK* mutations, 1 case with unspecified *ROS1* mutation. All 113 patients from the phase 2 trial had *ROS1* fusions.

No dose-limiting toxicities (DLTs) were observed in the dose-escalation portion of the phase I trial. Patients receiving unecritinib 300 mg BID reported lower rates of grade 3 or higher adverse events (AEs) and treatment-emergent AEs leading to dose reductions or interruptions than those receiving unecritinib 350 mg BID (Supplementary Table S2). Taking into consideration the results of subsequent efficacy analysis, we chose oral unecritinib 350 mg BID as the maximum-tolerated dose (MTD) and unecritinib 300 mg BID as the recommended phase II dose (RP2D) to provide a safe dosing regimen without jeopardizing the efficacy of unecritinib.

Grade 3 or higher TRAEs occurred in 41.0% of the patients in the phase I trial and 46.9% of the patients who received unecritinib 300 mg BID in both trials (Supplementary Tables S2 and S3). TRAEs led to dose reductions in 17.2% and dosing interruptions in 37.5% of the patients in all patients who received unecritinib 300 mg BID. Three patients (2.3%) discontinued unecritinib due to TRAEs. Among 128 patients who received RP2D 300 mg BID in both trials, the most frequently reported grade 3 or 4 TRAEs were reduced neutrophil count (25.8%), elevated alanine aminotransferase (7.8%), and reduced leukocyte count (6.3%) (Table 2). Reduced neutrophil count led to treatment interruptions in 26 cases (20.3%), but no treatment discontinuation. Treatment-related ocular disorders (cluster term) were reported in 28.1% of the patients, but none were grade 3 or higher (Supplementary Table 4). Furthermore, 34.4% of the patients had neurotoxicity (cluster term), and none were grade 3 or higher. TRAEs were manageable through dose reductions or interruptions and supportive therapy. No treatment-related death occurred in either trial. In addition, 4 patients in the phase II trial received prior PD-1/PD-L1 inhibitor therapy. They experienced a total of 88 any grade TRAEs, all of which were grade 1 or 2 except grade 3 elevated alanine aminotransferase in 1 patient.

**Table 2:** Treatment-related adverse events (TRAEs) ≥ 10% in patients treated with unecritinib 300 mg BID

	All (*n* = 128)	Grade 3 or higher
*Any TRAEs (≥10%)*^a^	126 (98.4)	60 (46.9)
Elevated aspartate aminotransferase	94 (73.4)	5 (3.9)
Elevated alanine aminotransferase	92 (71.9)	10 (7.8)
Vomiting	77 (60.2)	1 (0.8)
Reduced neutrophil count	71 (55.5)	33 (25.8)
Reduced leukocyte count	66 (51.6)	8 (6.3)
Sinus bradycardia	60 (46.9)	0 (0.0)
Diarrhea	55 (43.0)	0 (0.0)
Elevated creatine phosphokinase	50 (39.1)	4 (3.1)
Nausea	46 (35.9)	0 (0.0)
Neurotoxicity^b^	44(34.4)	0 (0.0)
Dysgeusia	17 (13.3)	0 (0.0)
Dizziness	14 (10.9)	0 (0.0)
Constipation	41 (32.0)	0 (0.0)
Elevated lactate dehydrogenase	41 (32.0)	0 (0.0)
Elevated creatine phosphokinase-MB	39 (30.5)	0 (0.0)
Ocular disorders^b^	36 (28.1)	0 (0.0)
Elevated plasma creatine	34 (26.6)	0 (0.0)
Hypoproteinemia	34 (26.6)	1 (0.8)
Anemia	33 (25.8)	2 (1.6)
Peripheral edema	29 (22.7)	0 (0.0)
Fatigue	24 (18.8)	1 (0.8)
Elevated α-hydroxybutyric dehydrogenase	22 (17.2)	0 (0.0)
Elevated alkaline phosphatase	21 (16.4)	0 (0.0)
Elevated γ-glutamyl transferase	18 (14.1)	2 (1.6)
Decreased appetite	18 (14.1)	0 (0.0)
Positive occult blood test	17 (13.3)	0 (0.0)
QT interval prolongation	17 (13.3)	6 (4.7)
Increased body weight	15 (11.7)	2 (1.6)
Proteinuria	15 (11.7)	0 (0.0)
Hypertriglyceridemia	14 (10.9)	2 (1.6)
Rash	14 (10.9)	1 (0.8)
Hypokalemia	<10	3 (2.4)
Reduced neutrophil percentage	<10	1 (0.8)
Hyponatremia	<10	1 (0.8)
Abnormal liver function	<10	1 (0.8)
Hepatic injury	<10	1 (0.8)
Drug-induced hepatic injury	<10	1 (0.8)
Bone marrow suppression	<10	1 (0.8)
Keratolysis plantare sulcatum	<10	1 (0.8)
Urinary tract infection	<10	1 (0.8)
Eczema	<10	1 (0.8)
Somatic pain	<10	1 (0.8)

Data are expressed in number (%)

^a^Listed are adverse events that were deemed by the investigators to be related to treatment and reported in at least 10% of 128 patients, including 3 patients receiving unecritinib 300 mg BID from the dose escalation phase and 12 patients receiving unecritinib 300 mg BID from the dose expansion phase of phase I trial, and 113 patients from phase II trial

^b^This item comprised a cluster of adverse events that may represent similar clinical symptoms or syndromes

### Pharmacokinetics

**Fig. 1:**
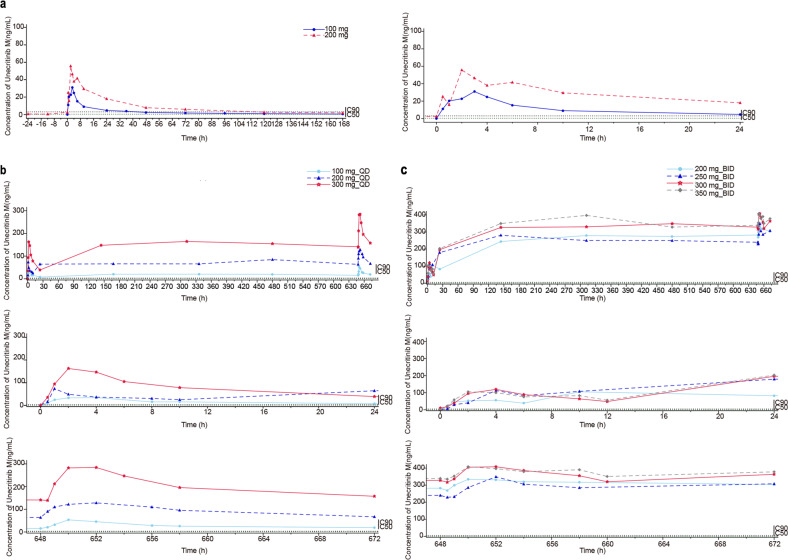
Pharmacokinetics characteristics. Mean plasma concentration-time curves of unecritinib M (**a**) after a single oral administration at 100 and 200 mg (linear scale). Mean plasma concentration-time curves of unecritinib M (**b**) after once daily multiple oral administrations at 100, 200, and 300 mg (linear scale). Mean plasma concentration-time curves of unecritinib M (**c**) after twice-daily multiple oral administrations at 200, 250, 300, and 350 mg (linear scale). The dotted lines indicate IC_50_ (0.8 nm [0.4 ng/mL], green) and IC_90_ (6.2 nm [2.8 ng/mL], black) of unecritinib M for wildtype ROS1

Unecritinib was rapidly absorbed regardless of doses or dosing schedule and reached peak plasma concentration in 30–60 min. Meanwhile, plasma unecritinib M concentrations peaked between 2 and 4 h after dosing (Fig. 1 and Supplementary Fig. S3). The levels of systemic exposure to unecritinib and unecritinib M (*C*_max_ and AUC_0–t_) increased as the dose level increased from 100 to 200 mg (single oral administration) and from 100 to 300 mg QD (multiple once-daily oral administrations). The dose proportionality was inconclusive for multiple twice daily oral administrations. Unecritinib had a seemingly dose-independent and rather brief *T*_1/2_ after single or multiple administrations. The short *T*_1/2_ caused no significant plasma accumulation of unecritinib over time, with a geometric mean Rac between 0.8 and 1 at the steady state after multiple twice-daily oral administrations at 200–350 mg. Though the geometric mean Rac ranged between 0.8 and 1.5 at the steady state after multiple once-daily oral administrations at 100, 200, and 300 mg, it was dose independent. Notably, the metabolite of unecritinib had a considerably longer *T*_1/2_ (between 4.6 and 69.9 h) after single or multiple administrations, which contributed to plasma accumulation of unecritinib M, with a geometric mean Rac between 3.6 and 6.0 at the steady state after multiple twice daily oral administrations at 200–350 mg. Overall, CL/F of unecritinib M declined over time after multiple twice-daily oral administrations at 200–350 mg.

### Efficacy

#### Overall efficacy

**Fig. 2:**
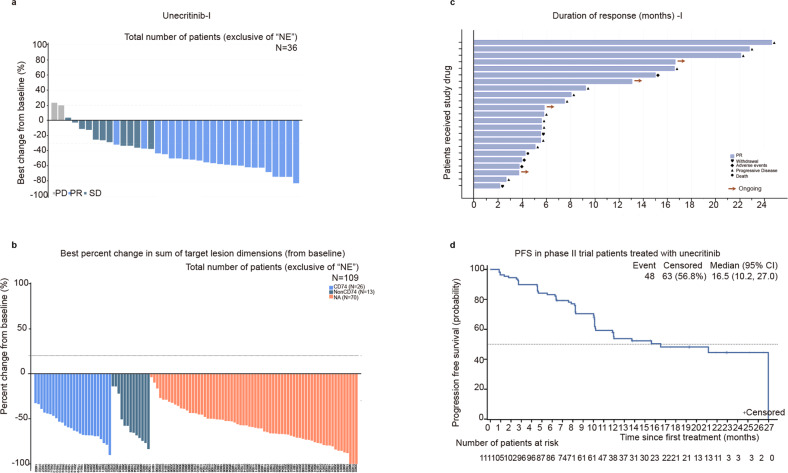
Treatment responses. Waterfall plots of the best percentage changes for the sum of target lesion diameters after unecritinib treatment are shown for individual patients with the best objective response assessed by the independent review committee (IRC) per RECIST v1.1 as indicated by the color codes. The dotted line indicates a 30% reduction in the target lesion size. Each bar represents one patient in the efficacy-evaluable population in phase I trial (**a**) and phase II trial (**b**). Treatment responses in (**b**) are also color-coded for gene fusion partners *CD74-ROS1*, non-*CD74-ROS1* fusions, and indeterminate. Swimmer plot of duration of treatment duration (months) of individual patients in phase I trial (**c**). Each bar represents one efficacy evaluable patient who had attained partial response. Deaths are marked in solid cycles. Patients receiving ongoing treatment are marked with an arrow. **d** The Kaplan–Meier curve for estimated progression-free survival (PFS) in the intention-to-treat population of phase II trial patients treated with unecritinib. Vertical lines on the survival curve indicate censoring of data. NE not evaluable

The ORR of the 36 efficacy evaluable patients in the phase I trial was 63.9% (95% CI 46.2–79.2) and the DCR was 94.4% (95% CI 81.3–99.3) (Fig. 2a, c; Supplementary Table S5).

At the data cutoff, 53 patients were still receiving unecritinib. Of the 113 patients who received unecritinib, 2 patients in the exploratory study cohort were excluded from the efficacy analysis. One of the 111 efficacy evaluable patients attained CR and 88 of them achieved PR, with an ORR of 80.2% (95% CI 71.5%, 87.1%). Furthermore, 9 patients had SD, and the DCR was 88.3% (95% CI 80.8%, 93.6%). Eleven patients had PD. One hundred and nine patients experienced a reduction from baseline in target lesion size (Fig. 2b). The median DOR was 20.3 months (95% CI 11.0, 26.1) (Table 3). Forty-eight PFS events had occurred and the median PFS was 16.5 months (95% CI 10.2, 27.0) (Fig. 2d). The 6-month PFS rate reached 83.2% (95% CI 74.6%, 89.0%) and the 12-month PFS rate 53.8% (95% CI 42.7%, 63.6%).

**Table 3:** Summary of efficacy endpoints

Efficacy	Efficacy-evaluable patients (*N* = 111)
*Best overall response, n (%)*	
CR	1 (0.9)
PR	88 (79.3)
SD	9 (8.1)
PD	11 (9.9)
NE	2 (1.8)
ORR, %	80.2
95% CI	71.5, 87.1
*DOR, months*	
Median	20.3
95% CI	11.0, 26.1
DCR, %	88.3
95% CI	80.8, 93.6
*PFS, months*	
Median	16.5
95% CI	10.2, 27.0
3 months, % (95% CI)	89.8 (82.4, 94.2)
6 months, % (95% CI)	83.2 (74.6, 89.0)
12 months, % (95% CI)	53.8 (42.7, 63.6)

Responses were evaluated per RECIST version 1.1. The Clopper–Pearson method was used for 95% CI. PFS was calculated using the Kaplan–Meier method

*CR* complete response, *DCR* disease control rate, *NE* not evaluable, *ORR* objective response rate, *PD* progressive disease, *PR* partial response, *SD* stable disease, *PFS* progression-free survival, *CI* confidence interval

#### Subgroup analysis

**Fig. 3:**
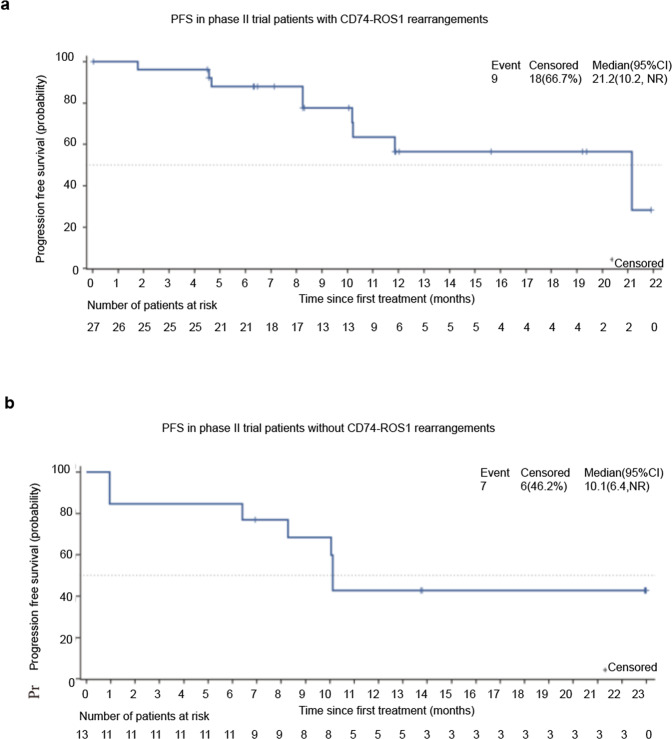
The Kaplan–Meier curve for estimated progression-free survival (PFS) in the intention-to-treat population of phase II trial patients treated with unecritinib, patients with (**a**) and without (**b**) *CD74-ROS1* rearrangements. Vertical lines on the survival curve indicate the censoring of data

Twenty-seven patients who had *CD74-ROS1* rearrangements exhibited a notable response to unecritinib therapy, with CR in 1 patient and PR in 23 patients, and an ORR of 88.9% (95% CI 70.8%, 97.7%). Furthermore, the median PFS was 21.2 months (95% CI 10.2, not reached) in patients with *CD74-ROS1* rearrangements (Fig. 3a) and 10.1 months (95% CI 6.4, not reached) in patients with non-*CD74-ROS1* rearrangements (Fig. 3b).

**Fig. 4:**
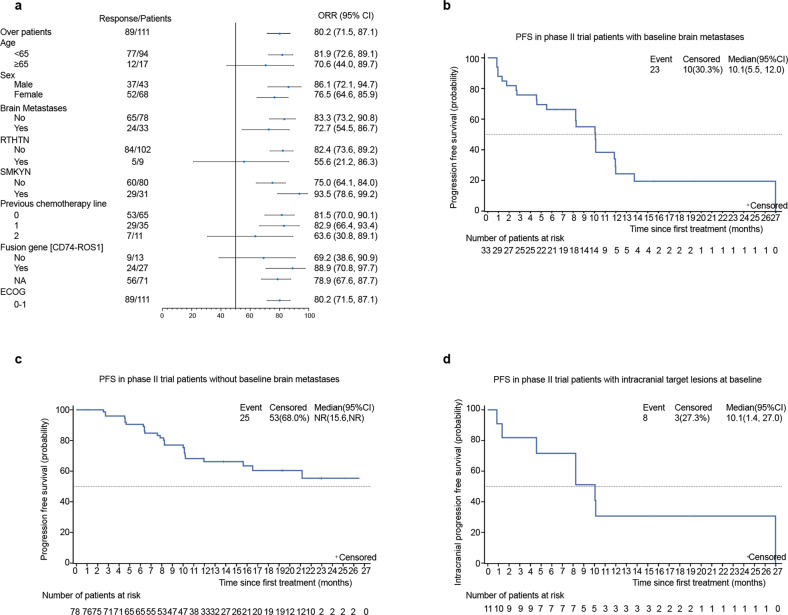
**a** Forest plot analysis of objective response rates (ORR) per patient subgroups in the main study cohort (*n* = 111). RTHYN, prior receipt of radiotherapy; SMKYN, prior or current smokers. The Kaplan–Meier curve for estimated PFS in the intention-to-treat population of phase II trial with (**b**) and without (**c**) baseline brain metastases who were treated with unecritinib. **d** The Kaplan Meier curve for estimated intracranial PFS. Vertical lines on the survival curve indicate the censoring of data

An exploratory subgroup analysis showed that both patients without brain metastasis (ORR 83.3%, 95% CI 73.2%, 90.8%) and those with brain metastasis (ORR 72.7%, 95% CI 54.5%, 86.7%) showed remarkable response to unecritinib therapy. Patients receiving no prior radiotherapy (ORR 82.4%, 95% CI 73.6%, 89.2%), prior or ever smoked (ORR 93.5%, 95% CI 78.6%, 99.2%), or no prior line of chemotherapy (ORR 81.5%, 95% CI 70.0%, 90.1%) exhibited significant response to unecritinib therapy. Patients receiving one prior line of chemotherapy also displayed notable responses to unecritinib therapy (ORR 82.9%, 95% CI 66.4%, 93.4%) (Fig. 4a). Twenty-three PFS events occurred among 33 patients with baseline brain metastases and the median PFS was 10.1 months (95% CI 5.5, 12.0) (Fig. 4b), with a 6-month PFS rate of 66.3% (95% CI 47.4%, 79.7%) and a 12-month PFS rate of 24.3% (95% CI 9.7%, 42.5%). Meanwhile, the median PFS was not reached among 78 patients without baseline brain metastases (Fig. 4c).

In addition, 4 patients (3 in the phase 1 trial and 1 in the exploratory study cohort of the phase 2 trial) had previously received crizotinib. Two of them attained PR as their best overall response (Supplementary Table S6).

#### Intracranial efficacy

There were 11 patients with intracranial target lesions among 33 patients who had brain metastases at baseline. Three patients had previously received radiotherapy for brain metastases. The intracranial ORR by IRC per RANO-BM was achieved in 8/11 patients (72.7%; 95% CI, 39.0%, 94.0%), including 1 patient who attained CR and 7 patients who achieved PR (Table 4). Two patients had SD, and 10/11 patients achieved disease control, with a DCR of 90.9% (95% CI, 58.7%, 99.8%). No patients developed PD. Eight (8/11, 72.7%) intracranial PFS events occurred and the median intracranial PFS was 10.1 months (95% CI 1.4, 27.0) (Fig. 4d). The 6-month PFS rate was 71.6% (95% CI 35.0%, 89.9%) and the 12-month PFS rate was 30.7% (95% CI 7.3%, 58.6%).

**Table 4:** Intracranial ORR, DCR, and PFS by independent review committee among patients with intracranial target lesions at baseline

Efficacy	Efficacy-evaluable patients (*N* = 11)
*Best overall response, n (%)*	
CR	1 (9.1)
PR	7 (63.6)
SD	2 (18.2)
PD	0 (0.0)
NE	1 (9.1)
ORR, %	72.7
95% CI	39.0, 94.0
DCR, %	90.9
95% CI	58.7, 99.8
*PFS, months*	
Median	10.1
95% CI	1.4, 27.0
3 months % (95% CI)	81.8 (44.7, 95.1)
6 months, % (95% CI)	71.6 (35.0, 89.9)
12 months, % (95% CI)	30.7 (7.3, 58.6)

Intracranial responses were assessed by the independent review committee per RANO-BM

The Clopper–Pearson method was used for 95% CI

PFS was calculated using the Kaplan–Meier method

*CR* complete response, *DCR* disease control rate, *DOR* duration of response, *NE* not evaluable, *ORR* objective response rate, *PD* progressive disease, *PR* partial response, *SD* stable disease, *PFS* progression free survival, *CI* confidence interval

## Discussion

Recent remarkable advances in molecular targeted therapies for advanced NSCLC harboring mutations such as *ALK* and *ROS1* rearrangement have extended the survival of these patients.^3–6,20^ However, safety concerns such as ocular toxicities with crizotinib.^12,16^ and neurotoxicity with entrectinib.^17^ have emerged, highlighting the need for the development of effective and safer molecular targeted therapeutic agents. We developed a novel TKI, unecritinib, with favorable pharmacokinetics characteristics and promising antitumor activities, which, at the same time, avoided or lessened the special toxicities of crizotinib and entrectinib. Our preclinical evidence indicates that this novel multi-kinase inhibitor of ROS1, ALK and c-MET has a greater bioavailability than crizotinib, and unecritinib M has a 200-fold lower distribution in ocular tissues than crizotinib. As far as we know, this is the largest trial of ROS-1 positive tumors harboring *ROS1* rearrangements showing that unecritinib had an acceptable toxicity profile, with lower rates of and no grade 3 or higher ocular toxicities and neurotoxicities, and elicited a durable response, with more than half (53.8%) of the patients being progression-free at 12 months. These findings demonstrate that unecritinib is safe and has promising activities against advanced NSCLC harboring *ROS1* rearrangement.

In this trial, unecritinib demonstrated promising antitumor activities in advanced NSCLC patients with *ROS1* rearrangement. Notably, unecritinib achieved an ORR by IRC of 80.2% while the ORR by IRC was 71.7% for crizotinib in the OO 12-01 study of East Asians with *ROS1*-positive advanced NSCLC^15^ and 67.1% for entrectinib in the integrated analysis of data from the STARTRK-1 and −2 and ALKA-372-001^21^ trials. The DCR reached 88.3% with unecritinib and is comparable to that of crizotinib (88.2%)^15^ and higher than that (75.8%) of entrectinib.^21^ The median PFS was 16.5 months in the current trial and 15.9 months with crizotinib^15^ and 15.7 months with entrectinib in the integrated analysis of data from the STARTRK-1 and -2 and ALKA-372-001^21^ trials. The high efficacy of unecritinib could be at least partially attributed to its high levels in the tumor tissues, as shown in our preclinical studies (data not published).

Previous studies showed that NSCLC patients with *ROS1* fusions are more predisposed to brain metastases^22^; 20–50% of NSCLC patients have brain metastases,^22^ and CNS is the site of initial progression in approximately half of *ROS1* fusion-positive NSCLC patients treated with crizotinib,^23^ highlighting the importance of CNS activity for ROS1 TKIs. There are no preclinical data on the comparison of the intracranial efficacy of unecritinib *versus* other ROS1 inhibitors.^24^ In the current study, the ORR of the patients who had brain metastases at baseline was 72.7% while that of entrectinib was 62.5%. In addition, unecritinib achieved an intracranial ORR of 72.7% per IRC among patients with baseline intracranial target lesions, which is largely comparable to that attained with entrectinib (79.2%) in the integrated analysis of data from the STARTRK-1 and -2 and ALKA-372-001^21^ trials. Notably, 46.4% of these patients in the integrated analysis had received radiotherapy for brain metastases, including 20 patients who received radiotherapy within the preceding 6 months and 26 patients who received or did not receive radiotherapy before the preceding 6 months. The intracranial ORR was 60% for those who had received radiotherapy within the preceding 6 months *versus* 46.2% for those who had not. Meanwhile, the intracranial PFS was 16.1 months for those who had received radiotherapy within the preceding 6 months *versus* 8.8 months for those who had not. Only 9 (8.1%) of our patients had received radiotherapy and 9.1% of the patients with baseline brain lesions had received radiotherapy. Meanwhile, ceritinib had a low intracranial ORR (29%) in NSCLC with *ROS1* rearrangements.^25^ Intracranial response to crizotinib was not assessed in the phase II OO 12-01 study of East Asians with *ROS1*-positive advanced NSCLC.^15^ and the intracranial ORR was 33% (2/6) with crizotinib in the phase II METROS trial.^13^ In addition, the median intracranial PFS reached 10.1 months in our patients with baseline intracranial target lesions *versus* 12.0 months with entrectinib in patients with measurable central nervous system lesions at baseline.^21^

Accurate delineation of targetable oncogenic alterations is critical to the targeted therapies for NSCLC^21^ and testing of all non-squamous NSCLC for therapy-predictive biomarkers is recommended in clinical practice guidelines.^26^ Our previous study showed that NSCLC patients with different *ROS1* fusions responded differently to crizotinib therapy.^22^ Among diverse *ROS1* fusions, *CD74* is the most frequent *ROS1* fusion partner and *CD74–ROS1* fusion is present in ~44% of NSCLC cases with *ROS1* rearrangements.^18,19^ In this trial, about two-thirds of the NSCLC patients (67.5%) who had undergone central NGS testing had *CD74-ROS1* fusion. Currently, it remains inconclusive whether TKI is efficacious for NSCLC harboring *CD74-ROS1* fusions. In the PROFILE 1001 trial, crizotinib efficacy was independent of *ROS1* translocation type.^14^ A retrospective analysis showed that patients whose tumor harboring *CD74-ROS1* had a significantly shorter median PFS with first-line crizotinib than those harboring non-*CD74-ROS1* fusions^27^ (17.0 months vs. 21.0 months; *P* = 0.008). Reduced efficacy of crizotinib in *CD74-ROS1*-positive patients was also noted by other investigators.^12,22^ The ORR with entrectinib for those with *CD74-ROS1* fusions was 72.9%, which is higher than that (67.1%) of the overall patient populations.^21^ In the current trial, unecritinib attained an ORR of 88.9% in patients with *CD74-ROS1* rearrangements, and the median PFS of these patients doubled that of patients without *CD74-ROS1* rearrangements (21.2 *vs*. 10.1 months). To our knowledge, this is the first time that ROS1-TKI has been found to have a greater ORR and longer PFS in a large sample of patients with *CD74-ROS1* rearrangements in a prospective clinical study.

The safety profile of unecritinib is overall consistent with that of other ROS1 inhibitors. The rate of drug dose interruptions due to TRAEs was 37.5% in the patients who received unecritinib 300 mg BID, which is higher than that (22.8%) of crizotinib.^15^ However, the proportion of patients who discontinued treatment due to TRAEs is low in these patients (2.3%) and comparable to that of crizotinib.^12^ Reduced neutrophil count was the most frequent TRAE in this study, occurring in 25.8% of the patients, compared to 12% with crizotinib.^12^ Reduced neutrophil count contributed to treatment interruptions in this trial, but it was manageable through dose reductions or interruptions and supportive therapy, which may explain the similar rate of treatment discontinuations of unecritinib and crizotinib. Among 128 patients who received unecritinib 300 mg BID, the other most frequently reported grade 3 or 4 TRAEs included elevated alanine aminotransferase (7.8%), and reduced leukocyte count (6.3%), which are common AEs that are also reported with other ROS1 TKIs. The TRK family of kinases are implicated in CNS homeostasis and inhibition of these kinases may lead to CNS symptoms such as dizziness and dysgeusia.^28^ Dizziness occurred in 10.9% and dysgeusia in 13.3% of our patients receiving unecritinib 300 mg BID, but neither was grade 3 or higher. These CNS abnormalities were also reported in NSCLC patients treated with crizotinib (dizziness 16%; dysgeusia 18%)^12^ and entrectinib (dizziness 34.8%; dysgeusia 43.4%).^21^ Ophthalmological AEs are unique AEs that were reported in a high proportion of NSCLC patients treated with crizotinib (82%)^12^ and also occurred in patients treated with other ROS1 inhibitors including brigatinib,^29^ entrectinib,^30^ and lorlatinib.^18^ As expected, ophthalmological AEs including visual impairment and blurred vision occurred in 36 patients (28.1%) in the current trial. This low rate of ophthalmological AEs might be at least partially due to the notably smaller distribution of unecritinib metabolite in ocular tissues than that of crizotinib, as shown in our preclinical studies (data not published). In addition, though the rate of any grade elevated AST (73.4%) and elevated ALT (71.9%) is higher with unecritinib than that reported for crizotinib (22% and 14%, respectively),^4^ the rate of grade III or higher elevated AST (3.9%) and ALT (7.8%) is largely comparable to crizotinib (2% and 4%, respectively).

A previous pharmacokinetics analysis of quantifiable unecritinib and unecritinib M data from 40 subjects after oral administration of unecritinib showed that unecritinib pharmacokinetics could be explained by a 1-compartment model with first-order absorption and elimination for unecritinib coupled with a 2-compartment model with time-dependent clearance for the metabolite.^31^ Our pharmacokinetics analysis found that the AUC_0_-t of unecritinib increased in a dose-proportional manner following rapid intestinal absorption of unecritinib BID from 200 to 350 mg. However, there was no apparent accumulation of unecritinib with unecritinib BID. The metabolite unecritinib M had considerable plasma accumulation at the steady state after multiple twice-daily oral administrations at 200–350 mg. Unecritinib is not a next-generation ROS1 inhibitor that could overcome ROS1 resistance mutations. Novel next-generation TKIs such as repotrectinib which has highly potent activities against ROS1, TRKA-C, and ALK could target on-target kinase domain mutations, which are a major mechanism of acquired resistance.^32^ In the early-stage TRIDENT-1 trial, repotrectinib demonstrated potent activities in *ROS1* rearranged NSCLC with solvent-front mutations.^33^ Taletrectinib, a novel ROS1 and pan-NTRK TKI, has demonstrated preclinical activities against *ROS1*^G2032R^ solvent-front mutation and also shown meaningful clinical antitumor activities against advanced crizotinib-refractory *ROS1* + NSCLC.^34^ The 4^th^ generation TKI NVL-655 with activities against a variety of ALK mutations is under active development.^35^ These novel TKIs could offer potentially effective treatment for NSCLC with solvent-front mutations including ALK^G1202R^, ROS1^G2032R^ or ROS1^D2033N^, TRKA^G595R^, and TRKC^G623R^. Two patients in our study received prior crizotinib and achieved PR. It is of intrigue and clinical relevance to learn whether unecritinib could also target ROS1 resistance mutations in advanced NSCLC, the answer to which, however, requires validation in future clinical trials with a larger target population.

Overall, this study demonstrated that unecritinib had an acceptable safety profile and established the clinical benefit of unecritinib for ROS1 inhibitor-naive patients with *ROS1-*positive advanced NSCLC and other selected advanced solid tumors who had failed prior chemotherapy. Notably, unecritinib was more effective in *CD74-ROS1* rearrangements NSCLC patients. Given that the CNS is a common site of metastases and a frequent site of disease progression in advanced NSCLC, unecritinib led to durable intracranial tumor responses, reflecting its potent intracranial activities. The findings strongly support that unecritinib should become one of the standards of care for NSCLC harboring *ROS1* rearrangements, particularly those patients with baseline brain metastases or *CD74-ROS1* fusions, and further clinical development of unecritinib for NSCLC with *ALK* rearrangement is warranted.

## Materials and methods

### Ethics statement

The trial protocols were approved by the independent ethics committee at each site and complied with the International Ethical Guidelines for Biomedical Research Involving Human Subjects. The studies were conducted according to Good Clinical Practice guidelines and the Declaration of Helsinki. All patients provided written informed consent prior to any trial activities.

### Screening criteria

This phase I/II trial enrolled adult patients with histologically confirmed advanced tumors (phase I) or locally advanced or metastatic NSCLC with *ROS1* rearrangements (phase II). *ROS1* rearrangements were determined using a reverse transcription-polymerase chain reaction (RT-PCR) AmoyDx assay.^15^ (Amoy Diagnostics, Xiamen, China) in the main study cohort of the phase II trial. Furthermore, *ROS1* fusions detected at the central laboratory underwent next-generation sequencing (NGS) for further comparison of efficacy for patients with *CD74-ROS1* fusions and those with non-*CD74-ROS1* fusions if there were enough tissue. NGS was performed on the NextSeq CN500 platform (Berry Genomics Co., Ltd) at a central laboratory using the HANDLE Classic NGS Panel (Amoy Diagnostics). Patients who were positive for *ROS1* fusions other than those included in the AmoyDx assays as detected by fluorescent in situ hybridization (FISH) or NGS and those who progressed on crizotinib were categorized into the exploratory study cohort of the phase II trial. Other eligibility criteria were no more than two prior chemotherapy regimens, at least one radiologically confirmed measurable target lesion per Response Evaluation Criteria in Solid Tumors (RECIST) version 1.1, and an Eastern Cooperative Oncology Group (ECOG) performance status (PS) of 0–1. Prior therapy with crizotinib or other ROS1 inhibitors was not permitted in the main study cohort. Additional eligibility criteria are provided in the study protocol.

### Study design and treatment

In the phase I trial, the MTD and the RP2D were determined using a dose–escalation strategy with a 3 + 3 design using a modified Fibonacci scheme, with preferential enrollment of *ALK* and *ROS1*-rearranged NSCLC patients. In the phase II trial, all patients harboring *ROS1* rearrangement received the RP2D of unecritinib 300 mg twice daily (BID) orally in continuous 28-day cycles until disease progression or development of unacceptable toxicity. The primary endpoints for phase I trial were to determine the safety, tolerability, DLTs, MTD, and pharmacokinetics of unecritinib. The primary endpoint of the phase II trial was ORR per the independent review committee (IRC). Secondary endpoints included the antitumor activities of unecritinib in ALK- and ROS1-rearranged advanced lung cancer patients for phase I and duration of response (DOR), disease control rate (DCR), PFS, intracranial ORR, intracranial DOR, intracranial time to progression (TTP), intracranial PFS, overall survival and safety for phase II.

Dose modification was allowed for grade 3 or higher adverse events (AEs) per the National Cancer Institute Common Terminology Criteria for AEs (NCI-CTC AE) version 5.0, with two levels of dose reduction, from 300 mg BID to 250 mg BID and from 250 mg BID to 200 mg BID. If more than two dose levels need to be reduced, the study treatment was terminated.

The trial protocols were approved by the independent ethics committee at each site and complied with the International Ethical Guidelines for Biomedical Research Involving Human Subjects. The studies were conducted according to Good Clinical Practice guidelines and the Declaration of Helsinki. All patients provided written informed consent prior to any trial activities.

### Assessments

Tumor responses were evaluated by investigators and IRC per RECIST v1.1 using computed tomography (CT) or magnetic resonance imaging (MRI) within 2 weeks of study entry, at 6 weeks post-treatment, and once every two cycles thereafter. Complete response (CR), partial response (PR), and stable disease (SD) had to be confirmed with a repeat scan after at least 6 weeks. The response of intracranial disease was evaluated radiologically per Response Assessment in Neuro-Oncology Brain Metastases (RANO-BM). AEs were evaluated using NCI-CTC AE version 5.0.

### Pharmacokinetic assessments

Blood samples for pharmacokinetics analyses were collected predose on days 1, 7, 14, 21, and 28, and 30 min, 1, 2, 4, 6, 10, and 12 h postdose on days 1 and 28. The concentrations of unecritinib and unecritinib M were measured by tandem mass spectrometry hyphenated to liquid chromatography separation systems (HPLC–MS/MS). The pharmacokinetics parameters were calculated by non-compartmental analysis (NCA) model using WinNonLin^®^ v 6.4 (Certara, Princeton, NJ, USA) and included *C*_max_ and time to reach *C*_max_ (*T*_max_), minimum and average steady-state concentration [*C*_SS(min)_ and *C*_ss-av_], the area under the steady-state plasma concentration–time curve (AUC_ss_), terminal elimination half-life (*T*_1/2_) and degree of fluctuation.

### Statistical analysis

Based on the phase II OO 12-01 study,^15^ an ORR of 50% as the lower limit of two-sided 95% CI was required to demonstrate the efficacy of unecritinib. The statistical power (1−*β*) to demonstrate efficacy based on this threshold was 85%. For a targeted ORR of 65%, with one-sided *α* of 0.025, 94 evaluable patients with *ROS1*-rearranged NSCLC were required for the main study cohort of the phase II trial. Assuming a drop-off rate of 15%, the maximum estimated target sample size was 111.

The Full Analysis Set (FAS) included all enrolled patients who had received at least one dose of unecritinib except for the patients enrolled into the exploratory study cohort, and the Per Protocol Set (PPS) included all enrolled patients who had received at least one cycle of treatment, with at least one radiological evaluation, good compliance, and no major study protocol violations. The pharmacokinetics concentration set included all enrolled patients who had received at least one dose of unecritinib and had one post-treatment measurement of plasma concentration of unecritinib. The pharmacokinetics parameter set includes all enrolled patients who had received at least one dose of unecritinib and had at least one valid pharmacokinetic parameter data. The safety set included all enrolled patients who had received at least one dose of unecritinib. No imputation was done for missing data. We estimated 95% CIs using the conventional Wald method for ORR and the Clopper-Pearson method for DCR and intracranial ORR. ORR and its 95% CI were presented graphically using forest plots. Kaplan–Meier methods were used to determine medians and 95% CIs for DOR, PFS, intracranial DOR, intracranial TTP and intracranial PFS. AEs were mainly analyzed using descriptive statistics.

Statistical analyses were undertaken using the SAS software package, version 9.4 M5 (SAS Institute Inc., Cary, NC). All tests were two-tailed with a level of significance set at *P* ≤ 0.05.

## Supplementary information

Study protocol

Supplementary Materials

## Data Availability

The datasets used and/or analyzed during the current study are available from the corresponding author on reasonable request.
