# Supplementary material for: Efficacy, safety and pharmacokinetics of Unecritinib (TQ-B3101) for patients with ROS1 positive advanced non-small cell lung cancer: a Phase I/II Trial
Source: Signal Transduct Target Ther. 2023 Jun 30;8:249. doi: 10.1038/s41392-023-01454-z (PMC10310851; doi:10.1038/s41392-023-01454-z)
Supplement: Supplementary file 1 — Study protocol [file 41392_2023_1454_MOESM1_ESM.docx]

Phase-I Clinical Trial Proposol for TQ-B3101

| **Title** | | Phase-I clinical trial for the tolerance and pharmacokinetics of TQ-B3101 |
| --- | --- | --- |
| **Sponsor** | | Chia Tai Tianqing Pharmaceutical Group Co., Ltd. |
| **Nature of study** | | Phase-I clinical trial of chemical medicine |
| **Objectives** | | Observe the tolerance of TQ-B3101 in advanced tumor patients to recommend the appropriate dose and administration method for the subsequent clinical trial.  Evaluate the pharmacokinetic characteristics of oral administration of TQ-B3101 capsules.  Preliminarily explore the effectiveness of ALK+ and ROS1+ in advanced lung cancer patients. |
| **Tolerance evaluation criteria** | | The NCI’s CTCAE 4.03 is used for evaluating the adverse reactions of the investigational medicine |
| **Safety indicators** | | Blood, urine, stool routine, biochemical tests (ALT, AST, TB, DB, BUN, Cr, and blood electrolytes), electrocardiogram, thyroid function, and coagulation function |
| **Study endpoint** | **Leading indicator** | Determine the dose-limiting toxicity (DLT) and maximum tolerated dose (MTD) of TQ-B3101 in tumor patients, and evaluate the pharmacokinetic characteristics of oral administration of TQ-B3101 |
|  | **Secondary indicator** | Preliminarily observe the antitumor efficacy of TQ-B3101 |
| **Study design** | | A single-center clinical trial of Phase I |
| **Number of cases included** | | 20 to 30 cases |
| **Principal investigator** | | Professor Hongming Pan |
| **Organization responsible for the clinical trial** | | Sir Run Run Shaw Hospital affiliated with the Zhejiang University School of Medicine |
| **Version No.** | | 3.0 |
| **Release date** | | July 10, 2018 |
| **Dosage regimen** | | - Dose escalation method of successive administration:   First administration: Abstain from eating but not drinking after 20:00 the day before the clinical trial. Take TQ-B3101 capsules orally on an empty stomach with 200 mL of warm water on the morning of the clinical trial day. Do not eat within one hour after taking the medicine.  Subsequent administration: Take the medicine orally once a day in the morning, and do not eat within one hour after administration. Twenty-eight days of continuous medication constitute one treatment cycle. For the patients on a twice-daily regimen, take the capsules orally on an empty stomach in the morning and evening, and abstain from eating within one hour after administration. During the clinical trial, abstain from tobacco, alcohol, coffee, carbonated drinks, and fruit juices, and avoid strenuous exercise.  With their prior consent, the patients deemed to benefit from the medicine in the follow-up study will continuously take the medicine until they are deemed by disease progression or investigators as no longer appropriate for continuous medication.  When new cases are no longer included, this clinical trial will be closed for patients under treatment if their minimum continuous medication period exceeds four cycles. For the patients under medication, the sponsor will continuously provide the medicine for free. |
| **Screening criteria** | | **Inclusion criteria:** Patients are eligible to be included in the clinical trial if they meet all of the conditions below:   1. An advanced malignant tumor patent definitively diagnosed by pathological and/or cytological examination has at least one measurable lesion; 2. Failure or relapse after conventional treatment, or patient refusal to accept an effective conventional treatment method; 3. Aged 18 to 70; ECOG performance status: 0 to 1; expected survival time: more than three months; 4. Major organs function normally; namely, the following criteria are met:    - Routine blood test: Hb ≥ 100g/L (no blood transfusion within 14 days); ANC ≥ 1.5×10^9^/L; PLT ≥ 100×10^9^/L    - Biochemical test: TBIL, ALT, AST, and serum Cr are within a normal range, or creatinine contour rate ≥ 60ml/min; triglyceride content ≤ 3.0mmol/L, and cholesterol content ≤ 7.75mmol/L;    - Doppler ultrasound evaluation: LVEF ≥ 50%. 5. Female patients should agree to take contraceptive measures (e.g., intrauterine devices [IUD], contraceptive pills, or condoms) during the clinical trial and within six months upon completion of the clinical trial. In addition, female patients should have a negative result in the serum or urine pregnancy test within seven days before inclusion in the clinical trial and must be non-lactating. Male patients should agree to take contraceptive measures during the study and within six months upon completion of the clinical trial. 6. Patients participate in the clinical trial voluntarily, sign an informed consent form, and comply well.   **Exclusion Criteria:** Patients should be excluded from the clinical trial if they meet one of the following conditions:   1. The patients had unresolved toxic reactions (except alopecia) of Grade 1 or above under CTCAE 4.03 due to previous treatment; 2. The patients had received systemic anti-cancer treatment, radiotherapy, or major surgery within two weeks before the clinical trial, received ALK or ROS1 inhibitor treatment within one week before the clinical trial, or participated in clinical trials of other medicines within four weeks; 3. The patients are subjected to diverse factors that affect the effectiveness of oral medication (e.g., inability to swallow, total gastrectomy, chronic diarrhea, and intestinal obstruction); 4. The patients with brain metastases have related symptoms, or their neurological symptoms are stably controlled for less than two weeks; 5. The patients suffer from suboptimal blood pressure control (systolic blood pressure > 140 mmHg, diastolic blood pressure > 90 mmHg), myocardial ischemia or myocardial infarction, cardiac arrhythmias (including QT interval ≥ 470 ms), heart rate of less than 60 bpm or bradycardia, and cardiac insufficiency of Grade I; 6. The patients suffer from fundus lesions; 7. The patients suffer from long-term unhealed wounds or fractures; 8. The patients show coagulation abnormalities, that is, bleeding tendencies (e.g., an active peptic ulcer) or are undergoing the thrombolytic or anticoagulant therapy; 9. The patients undergo arterial or venous thrombus events such as cerebrovascular accidents (e.g., temporary ischemic attacks) within 12 months before the first medication, or deep vein thrombosis or pulmonary embolism within six months before the first medication; 10. The patients have a history of psychotropic substance abuse, are unable to abstain from psychotropic substances, or suffer from a mental disorder; 11. The patients’ viral hepatitis B or hepatitis C are not stably controlled even after medicinal treatment (HBV - DNA ≥ 500copies); 12. The patients have a history of immunodeficiency (including HIV - positive results or other acquired or congenital immunodeficiency) or a history of organ transplantation; 13. According to the investigators’ judgment, the patients suffer from a severe concomitant disease that jeopardizes their safety or interferes with their ability to complete the clinical trial. |

# Phase-II Clinical Trial Proposol for TQ-B3101

| **No. of clinical trial proposal** | | TQ-B3101-II-01 |
| --- | --- | --- |
| **Title** | | Single-arm and multi-center clinical trial of Phase II for evaluating the efficacy and safety of the TQ-B3101 capsule monotherapy in patients with ROS1-positive non-small cell lung cancer |
| **Sponsor** | | Chia Tai Tianqing Pharmaceutical Group Co., Ltd. |
| **Registry classification** | | Chemical raw medicine Category 1.1 |
| **Objective** | | Evaluate the efficacy and safety of TQ-B3101 capsules in the treatment of ROS1-positive NSCLC patients |
| **Study design** | | A single-arm and multi-center clinical trial of Phase II |
| **Study subject** | | ROS1-positive locally advanced or metastatic NSCLC |
| **Sample size** | | 111 cases |
| **Principal investigator** | | Professor Shun Lu  Professor Hongming Pan |
| **Organization responsible for the clinical trial** | | Chest Hospital affiliated with the Shanghai Jiaotong University  Sir Run Run Shaw Hospital affiliated with the Zhejiang University School of Medicine |
| **Version No.** | | 3.0 |
| **Release date** | | February 17, 2020 |
| **Inclusion criteria:**  **Patients are eligible to be included in the clinical trial if they meet all of the conditions below:** | | 1. Gender: not limited; age (as of the date of signing the informed consent form): at least 18; 2. ECOG performance status: 0 to 1; expected survival time: more than three months; 3. The patients voluntarily participate in the clinical trial and sign an informed consent form; 4. Through histological or cytological examination, the patients are diagnosed as locally advanced or metastatic NSCLC (as per the staging criteria of the TNM Edition 8)   Note: If the test sample is pleural fluid, only the pathological results of pleural fluid embedding will be recognized;   1. The patients should provide a written report of ROS1-positive results (using NMPA - approved ROS1 RT-PCR test kits) at the screening stage. Alternatively, they should provide tumor histological specimens (including cell wax blocks) and biopsy tissue specimens obtained upon or after diagnosis before their inclusion in the trial study, and the specimens should be sent to the central laboratory for ROS1 test (RT - PCR test), with the results confirmed as ROS1-positive; 2. The patients have previously undergone not more than two chemotherapy regimens;   Note: If the patients have undergone more than two chemotherapy regimens, they should receive a biopsy again at the screening stage, and the results should be confirmed as ROS1 - positive (using NMPA - approved ROS1 RT - PCR test kits);   1. Within 28 days before the first administration of the medicine, there should be at least one evaluable target lesion other than a brain lesion confirmed by imaging examination (evaluated under the RECIST1.1 criteria) 2. Major organs function normally; namely, the following criteria are met:  - Routine blood test (no blood transfusion within 14 days): HB ≥ 90g/L; ANC ≥ 1.5×10^9^/L; PLT ≥ 100×10^9^/L; - Blood biochemistry:   TBIL ≤ 1.5 x ULN, ALT and AST ≤ 2.5 x ULN (in the absence of liver metastases) or ≤ 5 x ULN (in the presence of liver metastases);  Serum creatinine ≤ 1.5 × ULN; or calculated creatinine clearance rate ≥ 50 ml/min (using the Cockcroft - Gault formula); for male patients: CLcr = [(140 - age) × weight]/[72 × serum creatinine (mg/dL)]; for female patients: CLcr = 0.85 × CLcr of male patients).   - Doppler ultrasound: LVEF ≥ 50%.  1. Female patients must meet one of the following conditions:   ① Surgically sterilized;  ② Menopause for at least one year;  ③ The following conditions are met (if fertile):  Before inclusion in the clinical trial, the serum pregnancy test result is negative; they agree to use an approved method of contraception (e.g., oral contraceptive, injectable contraceptive or implanted, and shielding contraceptive methods such as spermicide and condoms, or an intrauterine device) during the entire study period until six months after the last administration.  Male patients must meet one of the following conditions:  ① Surgically sterilized;  ② Use an approved method of contraception during the entire study period until six months after the last administration. |
| **Exclusion criteria:**  **Patients should be excluded from the clinical trial if they meet one of the following conditions:** | | 1. The patients’ histological or cytological EGFR (+) is confirmed by previous test results or screening examination; 2. The patients have previously been treated with crizotinib or any other ROS1 inhibitor that is licensed or under clinical trial; 3. The patients are subjected to diverse factors that affect the effectiveness of oral medication (e.g., inability to swallow, total gastrectomy, chronic diarrhea, and intestinal obstruction); 4. The patients are suffering or have suffered from malignant tumors, excluding cured cervical carcinoma in situ, non-melanoma skin cancer, and superficial bladder tumor [Ta (non-invasive tumor), Tis (carcinoma in situ), and T1 (tumor-infiltrating basement membrane)] over the last five years; 5. The patients have a prior history of hypertensive critical phase, hypertensive encephalopathy, or uncontrolled hypertension (systolic blood pressure > 150 mmHg, or diastolic blood pressure > 100 mmHg after taking antihypertensive); 6. The patients suffer from clinically significant and uncontrolled cardiovascular disease, including but not limited to: QTc - interval abnormality (≥ 470 ms) or clinically significant ECG abnormality, arrhythmias requiring pharmacologic intervention (Grade 2 or above under CTCAE 5.0), and conduction problems; 7. The patients have undergone major surgery or antitumor treatment (e.g., chemotherapy, biological therapy, immunotherapy, traditional Chinese medicine treatment with antitumor indications, and radiotherapy for target lesions) within two weeks before this clinical trial or have participated in a clinical trial of other medicines during the past four weeks; 8. The patients suffer from known CNS metastases and/or spinal cord compression, carcinomatous meningitis, and soft meningeal disease, unless asymptomatic, or treated with stable curative effect (specifically, no imaging evidence of new brain metastases or enlarged brain metastases is available during at least two weeks after treatment of brain metastases, and steroid or anticonvulsant medication has been discontinued at least 14 days before the commencement of this clinical trial); 9. At the screening stage, the patients are confirmed as HCV - positive, HIV-positive, active syphilis positive, or HBsAg - positive, with the titer of HBV DNA of peripheral blood greater than the upper limit of its normal value and with a stable curative effect of fewer than 14 days after symptomatic treatment; alternatively, the patients have a history of stem cell or organ transplantation; 10. The patients have a history of psychotropic substance abuse, are unable to abstain from psychotropic substances, or suffer from a mental disorder; 11. According to the investigators’ judgment, the patients suffer from a severe concomitant disease that jeopardizes their safety or interferes with their ability to complete the clinical trial. |
| **Investigational product** | | Name: TQ-B3101  Dosage form: capsule  Specification: 50 mg, 100 mg, and 150 mg (subject to actual specification)  Manufacturer: Chia Tai Tianqing Pharmaceutical Group Co., Ltd.  Storage: Sealed in a lightproof environment and below 25 °C  Batch No.: Stated in the drug test report used during the clinical trial  Expiry date: Stated in the drug test report used during the clinical trial |
| **Dosage regimen** | | Based on the pharmacokinetic data in this clinical trial, the 300 mg bid dose group is selected for efficacy and safety evaluation.   1. Administration method:   300mg bid p.o.: Take the medicine in the morning and evening on an empty stomach, and preferably, abstain from eating within one hour before and after the administration; abstain from alcohol and tobacco, coffee, carbonated beverages, and fruit juice (e.g., citrus, peach, and grapefruit juice) during the clinical trial; and avoid strenuous exercise. Take the medicine for 28 consecutive days (i.e., one cycle) until the patients encounter disease progression or an adverse event of intolerance after treatment.   1. Dose adjustment:   If the patients experience an adverse event of Grade 3 or above under the NCI’s CTCAE 5.0, the dose should be adjusted once or several times at the discretion of the investigators.  Reduce dose for the first time:  Dosage regimen: 250 mg, bid, p.o.; Reduce dose for the second time:  Dosage regimen: 200 mg, bid, p.o.;  If the 200 mg bid dose level is still intolerable, the patients should terminate the clinical trial. |
| **Trial procedure** | | Screening stage (-28 to -1 day)  For the potential patients, the investigators should explain the benefits and risks of the clinical trial in detail. After they are fully informed and sign an informed consent form, they will undergo a screening examination first. If the investigators confirm that they meet the inclusion criteria and not the exclusion criteria, they are included in the clinical trial and are presented with the investigational medicine and log cards.  Treatment stage (days -3 to +3)  The patients should return to the hospital for a follow-up survey at the end of Cycle 1 (C1D28), Cycle 3 (C3D28), and Cycle 5 (C5D28), and every two cycles (eight visits) thereafter, focusing on concomitant medication, adverse events, laboratory examination, imaging examination, and life quality evaluation.  Non-PD survey (-7 to +7 day)  After disusing the investigational medicine for a non-progressive-disease (PD) reason, the patients should return to the clinical trial center for an end-of-treatment survey within 28 days after the last administration of the investigational medicine. Subsequently, the patients should receive a tumor evaluation every two cycles (days -7 to +7 over eight visits) until PD or until the patients start to receive other antitumor treatment. During the non-PD survey, the patients may receive vital signs and laboratory examinations as needed.  Survival follow-up period (days -7 to +7)  Upon completion of medicinal treatment, the investigators will survey the patients by phone every eight visits (days -7 to +7) to collect information on their subsequent antitumor treatment and survival until the patients die, withdraw the informed consent, or become out of contact, or the clinical trial is complete. |
| **Study endpoint** | **Leading indicator** | Objective remission rate (ORR) evaluated by an independent review committee |
|  | **Secondary indicator** | Indicators evaluated by an independent review committee, including the duration of remission (DOR), progression-free survival (PFS), disease control rate (DCR), and overall survival (OS), as well as intracranial remission rate (C - ORR), duration of intracranial remission (C - DOR), and time to intracranial disease progression (C - TTP) in connection with brain metastases |
| **Efficacy evaluation criteria** | | Efficacy is mainly evaluated in accordance with the RECIST 1.1, and the efficacy against brain metastases is evaluated in accordance with the RANO - BM criteria. |
| **Safety evaluation criteria** | | The adverse reactions are evaluated in accordance with the NCI’s CTCAE 5.0. |
| **Safety indicators** | | Record all clinical adverse events during the clinical trial and abnormalities in the laboratory examination, evaluate their correlation to the investigational medicine, and judge the severity of the adverse events. |
| **Statistical analysis** | | The SAS 9.4 software is used. All statistical tests are two-sided tests, and p ≤ 0.05 indicates that the tested differences are statistically significant, and a 95% confidence interval is used. |
| **Schedule** | | Planned start date: March 2019  Planned end date: March 2021 |
