# Supplementary material for: Efficacy, safety and pharmacokinetics of Unecritinib (TQ-B3101) for patients with ROS1 positive advanced non-small cell lung cancer: a Phase I/II Trial
Source: Signal Transduct Target Ther. 2023 Jun 30;8:249. doi: 10.1038/s41392-023-01454-z (PMC10310851; doi:10.1038/s41392-023-01454-z)
Supplement: Supplementary file 2 — Supplementary Materials [file 41392_2023_1454_MOESM2_ESM.docx]

Supplementary Materials for

**Efficacy, Safety and Pharmacokinetics of Unecritinib (TQ-B3101) for Patients with *ROS1* Positive Advanced Non-small Cell Lung Cancer: A Phase I/II Trial**

Shun Lu^1†*^, Hongming Pan^2†*^, Lin Wu^3^, Yu Yao^4^, Jianxing He^5^, Yan Wang^6^, Xiuwen Wang^7^, Yong Fang^2^, Zhen Zhou^1^, Xicheng Wang^8^, Xiuyu Cai^9^, Yan Yu^10^, Zhiyong Ma^11^, Xuhong Min^12^, Zhixiong Yang^13^, Lejie Cao^14^, Huaping Yang^15^, Yongqian Shu^16^, Wu Zhuang^17^, Shundong Cang^18^, Jian Fang^19^, Kai Li^20^, Zhuang Yu^21^, Jiuwei Cui^22^, Yang Zhang^23^, Man Li^23^, Xinxuan Wen^24^, Jie Zhang^25^, Weidong Li^26^, Jianhua Shi^27^, Xingxiang Xu^28^, Diansheng Zhong^29^, Tao Wang^30^, Jiajia Zhu^3^

Correspondence to: Shun Lu (shunlu@sjtu.edu.cn), Hongming Pan (panhongming@zju.edu.cn)

**This file includes:**

Supplementary data;

Supplementary Figures S1 to S3;

Supplementary Tables S1 to S6;

ALL original and uncropped films of Western blots.

**Other Supplementary Materials for this manuscript include the following:**

Study protocol “Phase-I Clinical Trial Proposol for TQ-B3101 (Phase-I clinical trial for the tolerance and pharmacokinetics of TQ-B3101)” and “Phase-II Clinical Trial Proposol for TQ-B3101 (Single-arm and multi-center clinical trial of Phase II for evaluating the efficacy and safety of the TQ-B3101 capsule monotherapy in patients with ROS1-positive non-small cell lung cancer)”.

**Supplementary Data**

***Treatments***

In the dose-escalation phase of the phase I trial, sequential patient groups of up to 3 patients per dose level received unecritinib 100 mg, 200 mg, and 300 mg once daily (QD), and unecritinib 200 mg, 250 mg, 300 mg, and 350 mg twice daily (BID) orally in continuous 28-day cycles in a 3+3 design using a modified Fibonacci scheme, with the intention of determining the maximum-tolerated dose (MTD), defined as the highest dose level for which the incidence of dose-limiting toxicities (DLTs) was < 33%. DLTs including grade 3 or higher non-hematologic toxicity, grade 4 hematoxicity or grade 3 neutropenia (absolute neutrophil count < 1000/μL) with fever ≥ 38.5°C were determined based on the incidence and intensity of adver events (AEs) occurring up to 4 weeks after an unecritinib dose. The dose expansion portion had a planned enrollment of 8 to 20 patients.

In the phase II trial, patients received unecritinib 300 mg BID orally in continuous 28-day cycles. Treatment cycles were repeated until disease progression, unacceptable toxicity, or withdrawal of consent.

Best supportive care was provided. Patients were allowed to receive bisphosphonates for bone metastasis and palliative radiotherapy was allowed for uncontrollable metastasis-associated pain with irradiation field confined to less than 5% of the bone marrow.

***Assessments***

In the phase I trial, tumor responses were evaluated according to Response Evaluation Criteria in Solid Tumors (RECIST) version 1.1. Patients who had complete response (CR), partial response (PR) or stable disease (SD) after completion of two cycles of unecritinib may continue treatment upon agreement by both the investigator and the patient. CR or PR had to be confirmed no less than 4 weeks and SD 6-8 weeks after an initial response.

In the phase II trial, the patients underwent baseline tumor imaging, with computed tomography (CT) or magnetic resonance imaging (MRI) of the head, enhanced CT scan of the neck, chest, abdomen, and pelvis and whole-body bone scan. Responses were evaluated by investigators and an independent review committee (IRC) per RECIST version 1.1 using CT or MRI within 2 weeks of study entry, at 6 weeks post treatment and once every two cycles thereafter. CR, PR, and SD had to be confirmed with a repeat scan after at least 6 weeks. ORR was the percentage of patients with a best overall response of confirmed CR or PR and disease control rate (DCR) was the percentage of patients with confirmed CR, PR, or SD. Duration of response (DOR) was the time from initial CR or PR to PD or death, whichever occurred earlier. Patients were followed up every 8 weeks by telephone calls for data on survival and subsequent therapy until loss to follow up, death, or the end of the study. Progress free survival (PFS) was calculated from the date of the start of medication to PD or death, whichever occurred earlier. Overall survival was the time from the start of medication to death.

Intracranial ORR was the proportion of patients who attained intracranial CR or PR among patients with baseline intracranial target lesions. Intracranial DOR was the duration of intracranial response among patients with an intracranial response and intracranial time to tumor progression (TTP) was the time from the start of medication to intracranial disease progression. Intracranial PFS was calculated from the date of the start of medication to intracranial PD or death, whichever occurred earlier.


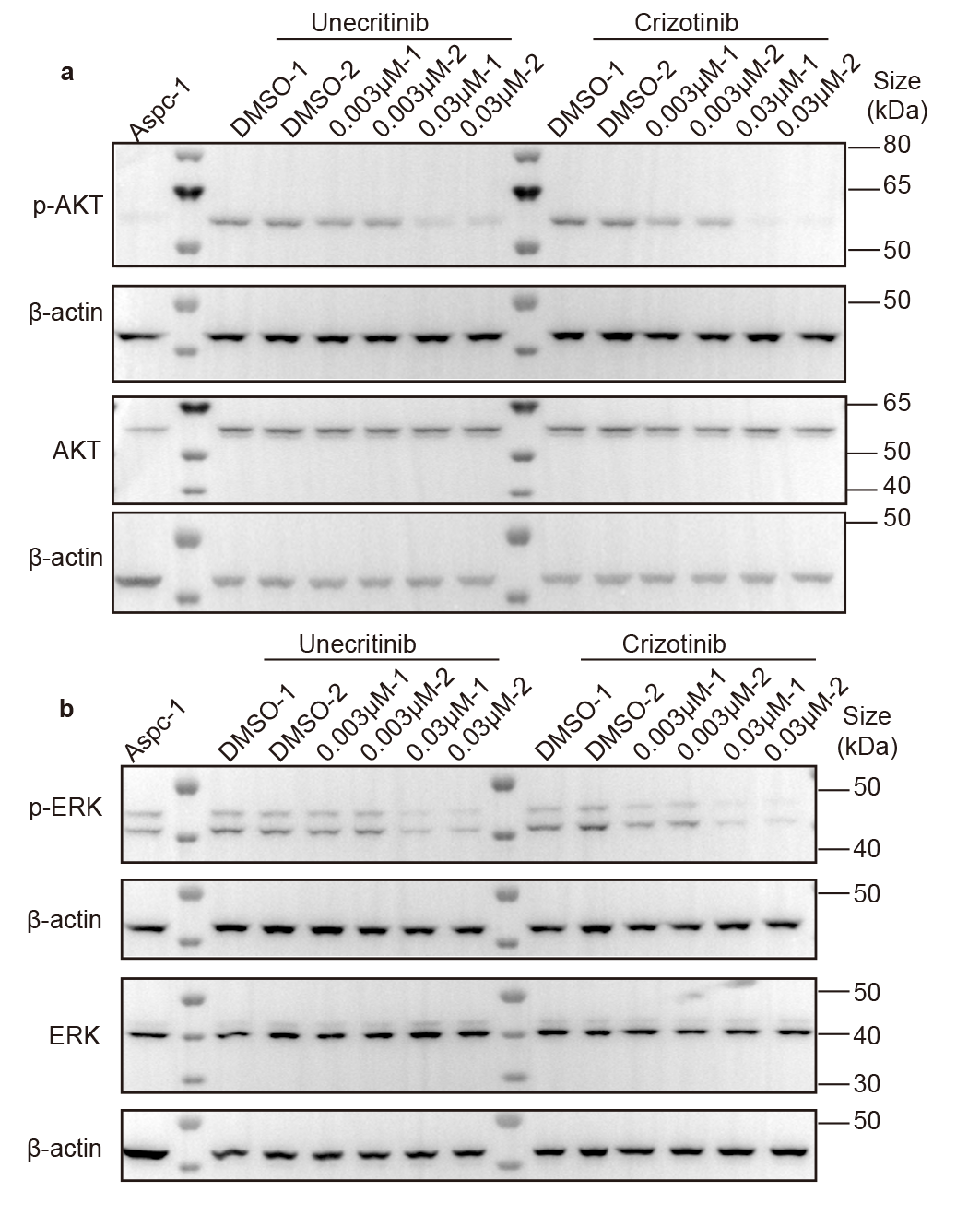


**Supplementary Figure S1** Unecritinib causes a similar inhibition of AKT phosphorylation (a) and its downstream signaling molecules ERK1/2 (extracellular signal-regulated protein kinases 1 and 2) to crizotinib (b). NSCLC HCC78 cells were treated with dimethyl sulfoxide (DMSO), unecritinib or crizotinib at the indicated concentrations. Immunoblotting assays were performed using antibodies against phospho-AKT, AKT, phosphor-ERK, ERK, and β actin. Pancreatic cancer Aspc-1 cells serve as positive control and β actin serves as loading control. Representative immunoblotting images are shown.


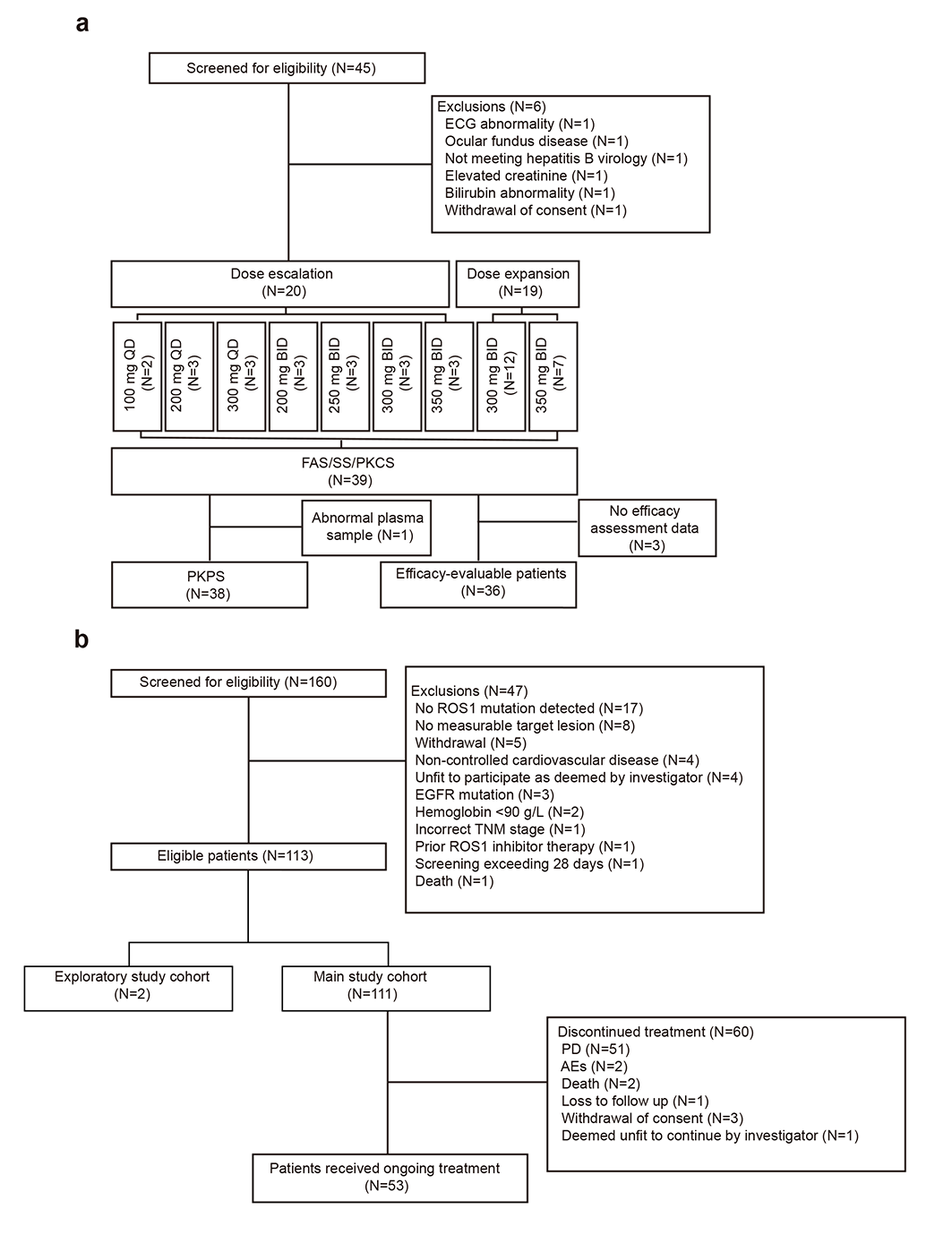


**Supplementary Figure S2.** The study flowchart. BID, twice daily; ECG, electrocardiogram; FAS, Full Analysis Set; PKCS, Pharmacokinetics Concentration **Set**; QD, once daily; PKPS, **Pharmacokinetics** Parameter **Set; SS, Safety Set.**

**
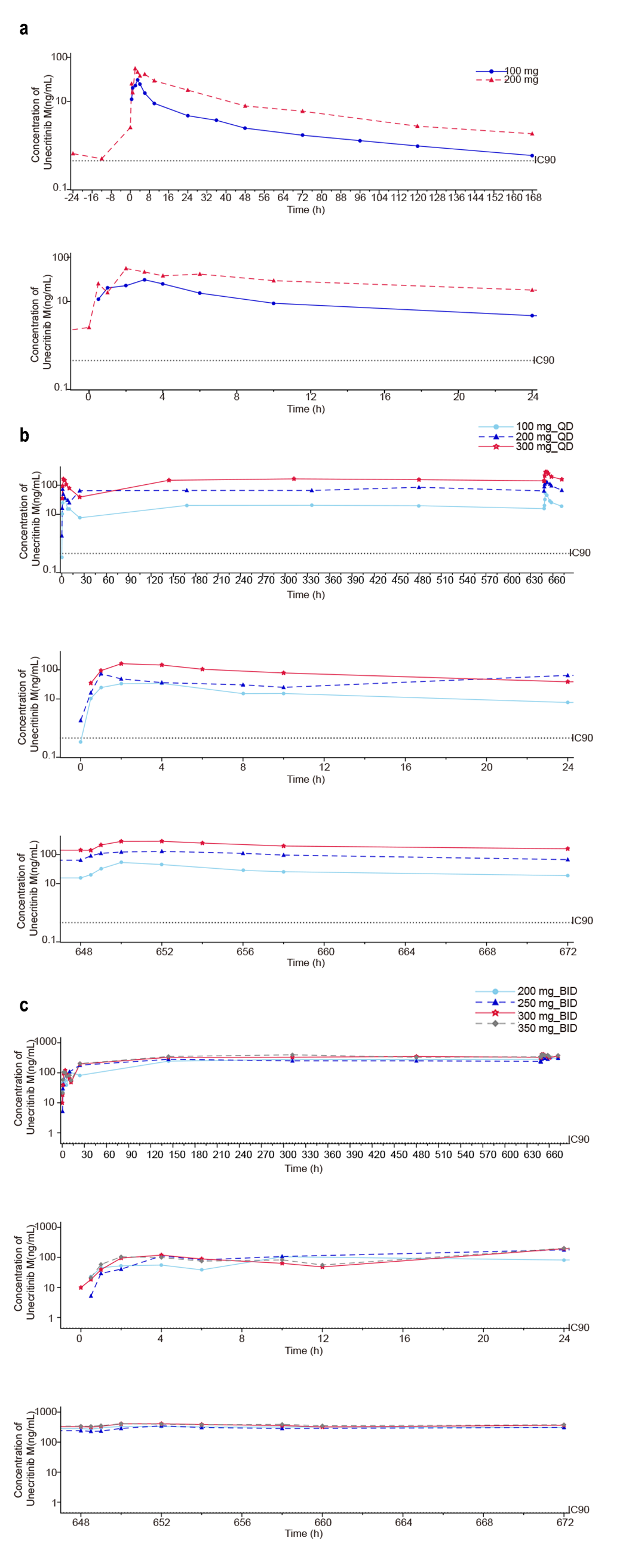
**

**Supplementary Figure S3.** Pharmacokinetics characteristics. Mean plasma concentration-time curves of unecritinib M (a) after a single oral administration at 100, and 200 mg ( semi-logarithmic scale). Mean plasma concentration-time curves of unecritinib M (b) after once daily multiple oral administrations at 100, 200, and 300 mg (semi-logarithmic scale). Mean plasma concentration-time curves of unecritinib M (c) after twice daily multiple oral administrations at 200, 250, 300 and 350 mg (semi-logarithmic scale). The dotted lines indicate IC50 (0.8 nm [0.4 ng/mL], green) and IC90 (6.2 nm [2.8 ng/mL], black) of unecritinib M for wildtype ROS1.

**Supplementary Table S1.** IC_50_ of unecritinib and crizotinib.

| IC50  (nM) | Lung cancer cell  NCI-H3122 | Lung cancer cell  NCI-H2228 | | Gastric cancer cell  SNU-5 |
| --- | --- | --- | --- | --- |
| Unecritinib | 180 | 378.9 | 20.3 | |
| Crizotinib | 213.5 | 358.5 | 29.1 | |

**Supplementary Table S2.** Summary of treatment-related adverse events in phase I trial.

|  | 100 mg QD  (N=2) | 200 mg QD  (N=3) | | 300 mg QD  (N=3) | 200 mg BID  (N=3) | 250 mg BID  (N=3) | 300 mg BID  (N=15) | 350 mg BID  (N=10) | All patients  (N=39) |
| --- | --- | --- | --- | --- | --- | --- | --- | --- | --- |
| TRAE | 2 (100.0) | 3 (100.0) | 2 (66.7) | | 3 (100.0) | 3 (100.0) | 15 (100.0) | 10 (100.0) | 38 (97.4) |
| ≥ 3 grade TRAE | 1 (50.0) | 0 (0.0) | 2 (66.7) | | 1 (33.3) | 0 (0.0) | 6 (40.0) | 6 (60.0) | 16 (41.0) |
| SAE | 0 (0.0) | 1 (33.3) | 0 (0.0) | | 1 (33.3) | 0 (0.0) | 4 (26.7) | 3 (30.0) | 9 (23.1) |
| TRAEs leading to dose reductions | 0 (0.0) | 0 (0.0) | 0 (0.0) | | 1 (33.3) | 0 (0.0) | 2 (13.3) | 3 (30.0) | 6 (15.4) |
| TRAEs leading to treatment interruptions and discontinuation | 0 (0.0) | 0 (0.0) | 0 (0.0) | | 1 (33.3) | 0 (0.0) | 6 (40.0) | 5 (50.0) | 12 (30.8) |
| TRAEs leading to death | 0 (0.0) | 0 (0.0) | 0 (0.0) | | 0 (0.0) | 0 (0.0) | 0 (0.0) | 0 (0.0) | 0 (0.0) |

TRAE, treatment-related adverse events

**Supplementary Table S3**. Summary of treatment-related adverse events in patients who received unecritinib 300 mg BID.

|  | Unecritinib 300 mg (N=128)* | |
| --- | --- | --- |
|  | All | ≥ Grade 3 |
| TRAEs | 126 (98.4) | 60 (46.9) |
| TRAEs leading to dose reductions | 22 (17.2) | 17 (13.3) |
| TRAEs leading to treatment interruptions | 48 (37.5) | 37 (28.9) |
| TRAEs leading to treatment discontinuation | 3 (2.3) | 2 (1.6) |
| TRAEs leading to death | 0 (0.0) | 0 (0.0) |

TRAE, treatment-related adverse events.

* Among 128 patients who received unecritinib 300 mg BID, 3 patients were from the dose escalation phase and 12 patients were from the dose expansion phase of phase I trial, and 113 patients were from phase II trial.

Data are expressed in number (%).

**Supplementary Table S4.** Ocular toxicities in the safety population (n=128), n (%).

| **Treatment-related ocular adverse events** | **Grade 1 or 2** | **Grade 3 or higher** |
| --- | --- | --- |
| **All** | 36 (28.1) | 0 |
| **Visual disturbances** | 12 (9.4) | 0 |
| **Blurred vision** | 8 (6.3) | 0 |
| **Diplopia** | 5 (3.9) | 0 |
| **Dry eyes** | 3 (2.3) | 0 |
| **Cataract** | 3 (2.3) | 0 |
| **Visual fatigue** | 1 (0.8) | 0 |
| **Flash hallucinations** | 1 (0.8) | 0 |
| **Arteriosclerotic retinopathy** | 1 (0.8) | 0 |
| **Vitreous opacities** | 1 (0.8) | 0 |
| **Retinal vascular disease** | 1 (0.8) | 0 |
| **Ocular cholesterolosis** | 1 (0.8) | 0 |
| **Eyelid edema** | 1 (0.8) | 0 |
| **Orbital edema** | 1 (0.8) | 0 |
| **Ocular pain** | 1 (0.8) | 0 |
| **Ocular degenerative disease** | 1 (0.8) | 0 |

**Supplementary Table S5.** Summary of ORR and DCR in the phase I trial.

| Efficacy | 100 mg QD  (N=2) | 200 mg QD  (N=3) | 300 mg QD  (N=3) | 200 mg BID  (N=3) | 250 mg BID  (N=3) | 300 mg BID  (N=14) | 350 mg BID  (N=8) | All patients  (N=36) |
| --- | --- | --- | --- | --- | --- | --- | --- | --- |
| Best overall response, n (%) | | | | | | | | |
| CR | 0 (0.0) | 0 (0.0) | 0 (0.0) | 0 (0.0) | 0 (0.0) | 0 (0.0) | 0 (0.0) | 0 (0.0) |
| PR | 1 (50.0) | 0 (0.0) | 1 (33.3) | 3 (100.0) | 2 (66.7) | 9 (64.3) | 7 (87.5) | 23 (63.9) |
| SD | 1 (50.0) | 2 (66.7) | 1 (33.3) | 0 (0.0) | 1 (33.3) | 5 (35.7) | 1 (12.5) | 11 (30.6) |
| PD | 0 (0.0) | 1 (33.3) | 1 (33.3) | 0 (0.0) | 0 (0.0) | 0 (0.0) | 0 (0.0) | 2 (5.6) |
| ORR, %  (95% CI) | 50.0  (1.3, 98.7) | 0.0  (0.0, 70.8) | 33.3  (0.8, 90.6) | 100.0  (29.2, 100.0) | 66.7  (9.4, 99.2) | 64.3  (35.1, 87.2) | 87.5  (47.4, 99.7) | 63.9  (46.2, 79.2) |
| DCR, %  (95% CI) | 100.0  (15.8, 100.0) | 66.7  (9.4, 99.2) | 66.7  (9.4, 99.2) | 100.0  (29.2, 100.0) | 100.0  (29.2, 100.0) | 100.0  (76.8, 100.0) | 100.0  (63.1, 100.0) | 94.4  (81.3, 99.3) |

Abbreviations: CR, complete response; DCR, disease control rate; NE, not evaluable; ORR, objective response rate; PD, progressive disease; PR, partial response; SD, stable disease.

Responses were evaluated per RECIST version 1.1.

The Clopper–Pearson method was used for 95% CI.

**Supplementary Table S6.** Treatment and best overall response of patients previously treated with crizotinib.

| **Subject No.** | **Sex/age** | **Treatment** | **BOR/Causes of treatment termination** | **Prior therapy** |
| --- | --- | --- | --- | --- |
| **TQ-B3101-1-0001-01-003** | M/64 | Unecritinib 200 mg QD | PD/PD | Crizotinib |
| **TQ-B3101-1-0001-01-023** | M/31 | Unecritinib 350 mg BID | PR/PD | Crizotinib |
| **TQ-B3101-1-0001-01-028** | M/65 | Unecritinib 350 mg BID | PR/AE | Crizotinib |
| **TQ-B3101-II-01-21-002** | M/53 | Unecritinib 300 mg | Death | Crizotinib |

Abbreviations: AE, adverse event; BOR, best overall response; PD, progressive disease; PR, partial response.

ALL original and uncropped films of Western blots.

**Supplementary Figure S1a: p-AKT**

**
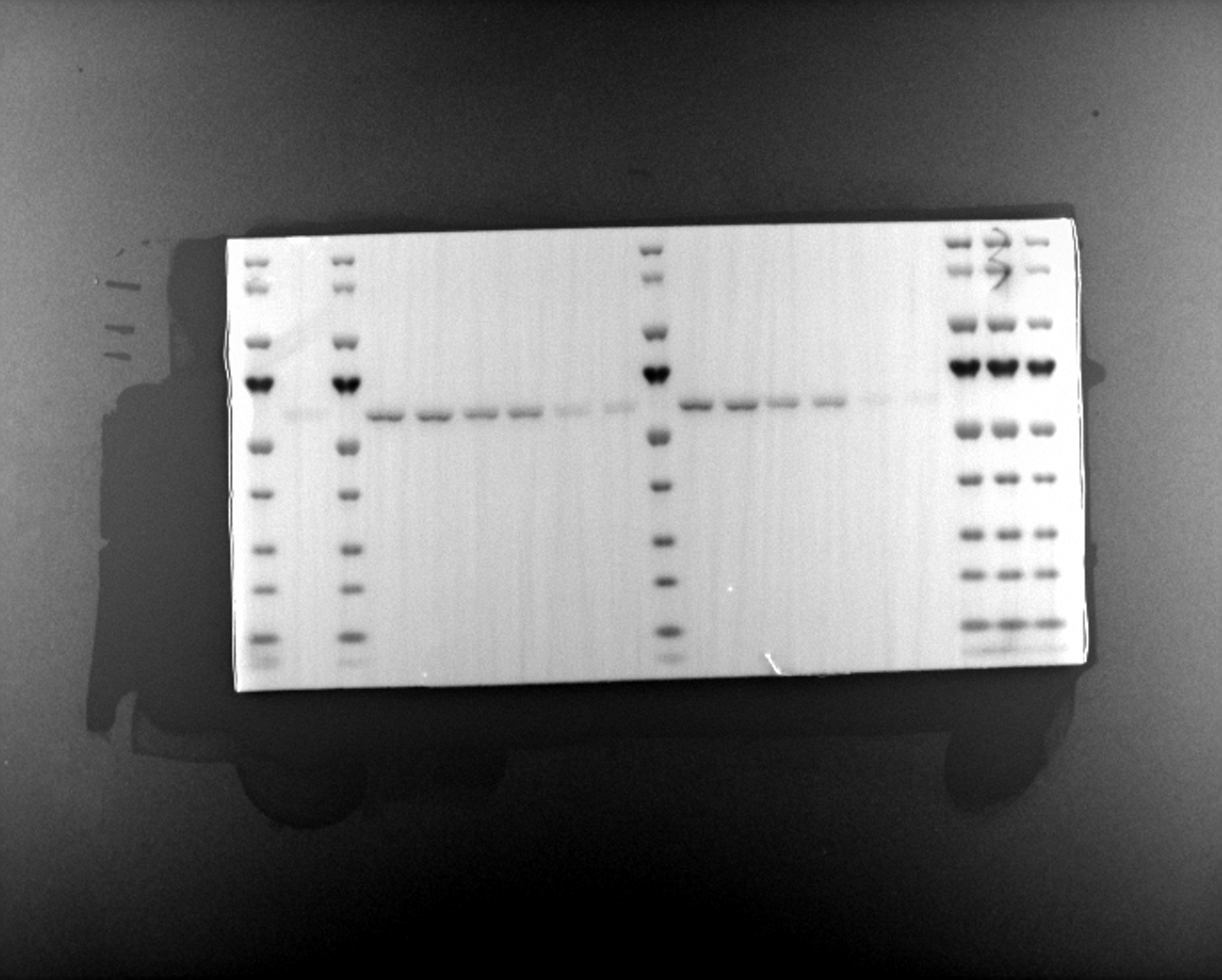
**

**— 65 kDa**

**Supplementary Figure S1a: p-AKT-β-actin**

**
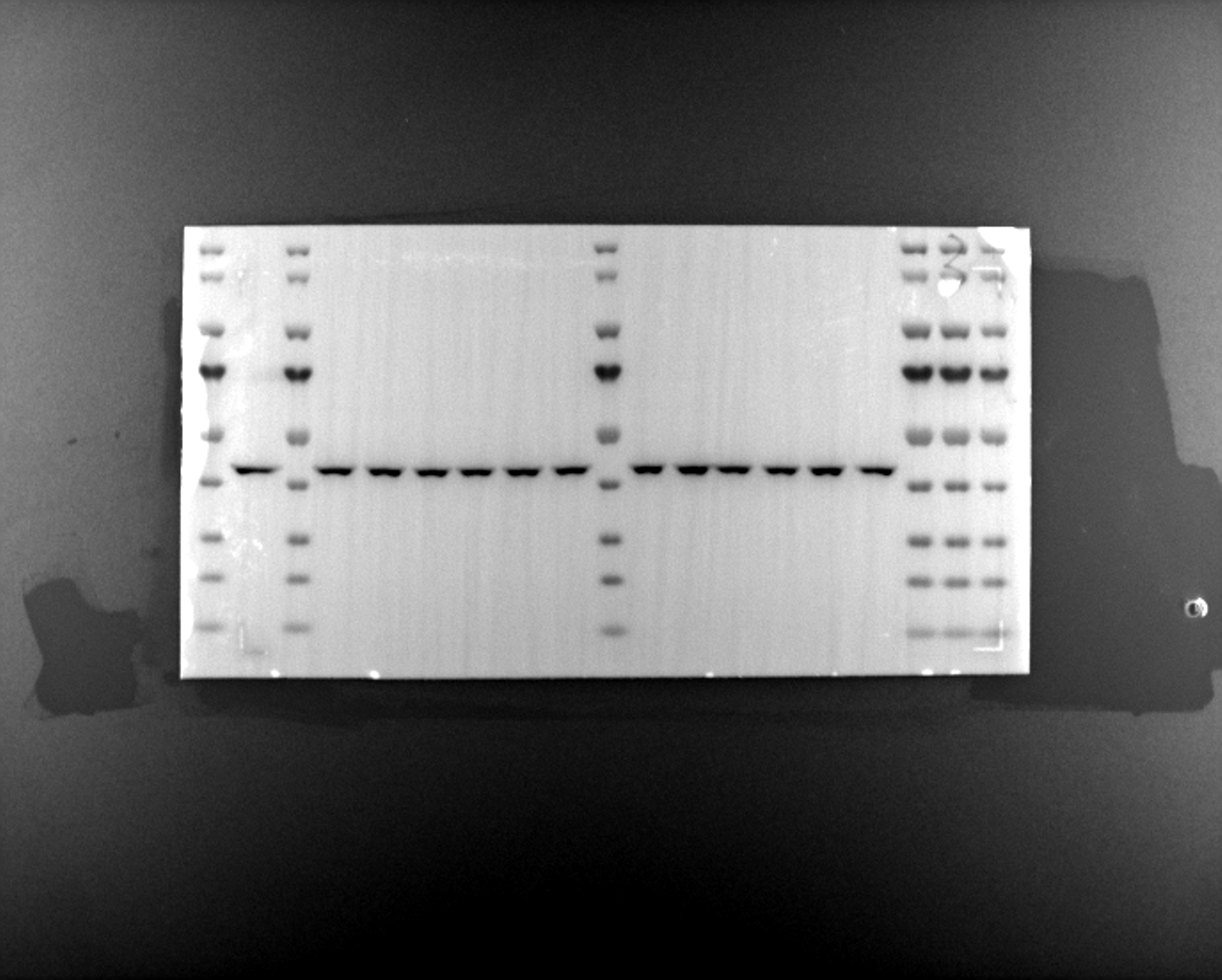
**

**— 50 kDa**

**Supplementary Figure S1a: AKT**


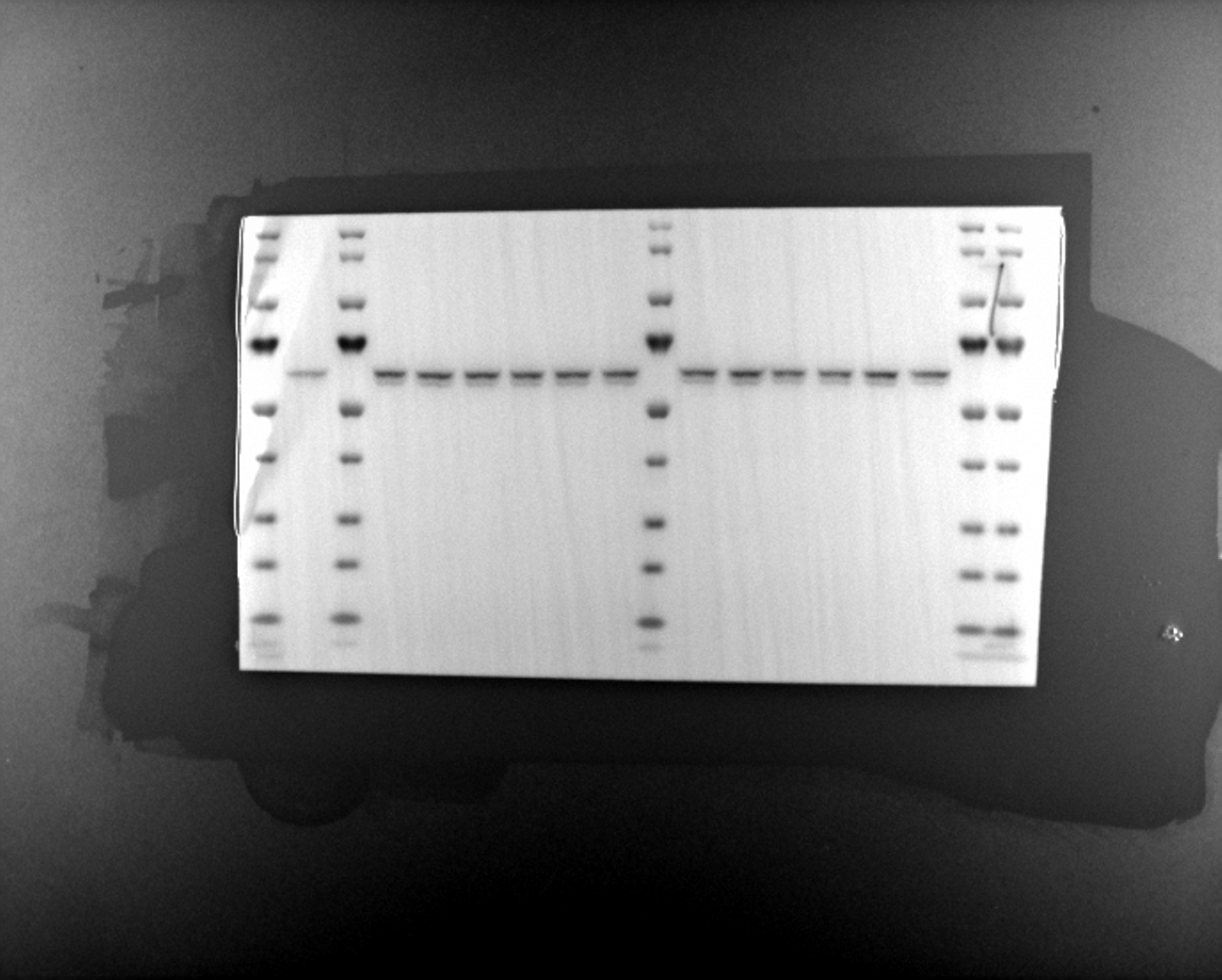


**— 65 kDa**

**Supplementary Figure S1a:** **AKT-β-actin**

**
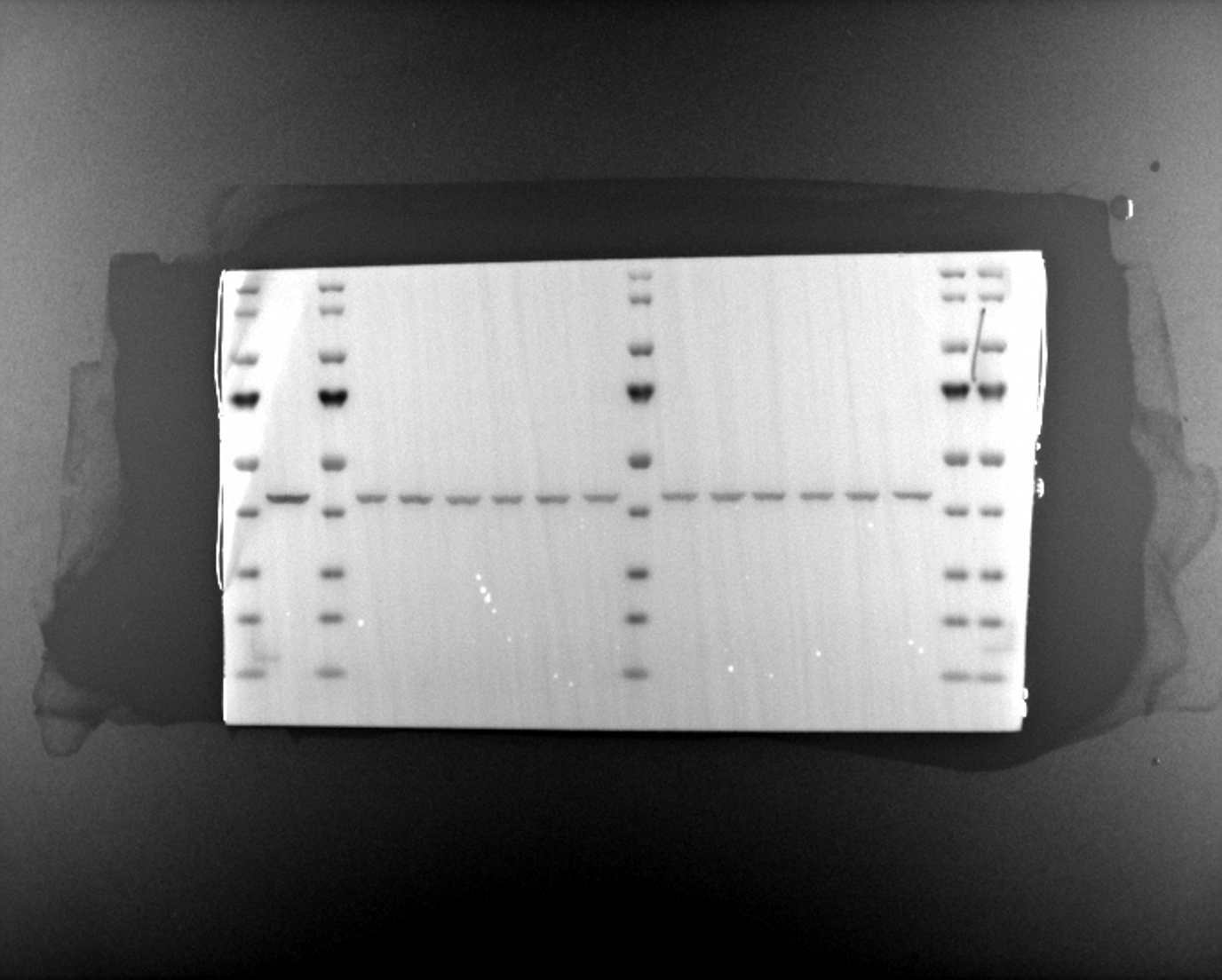
**

**— 50 kDa**

**Supplementary Figure S1b: p-ERK**

**
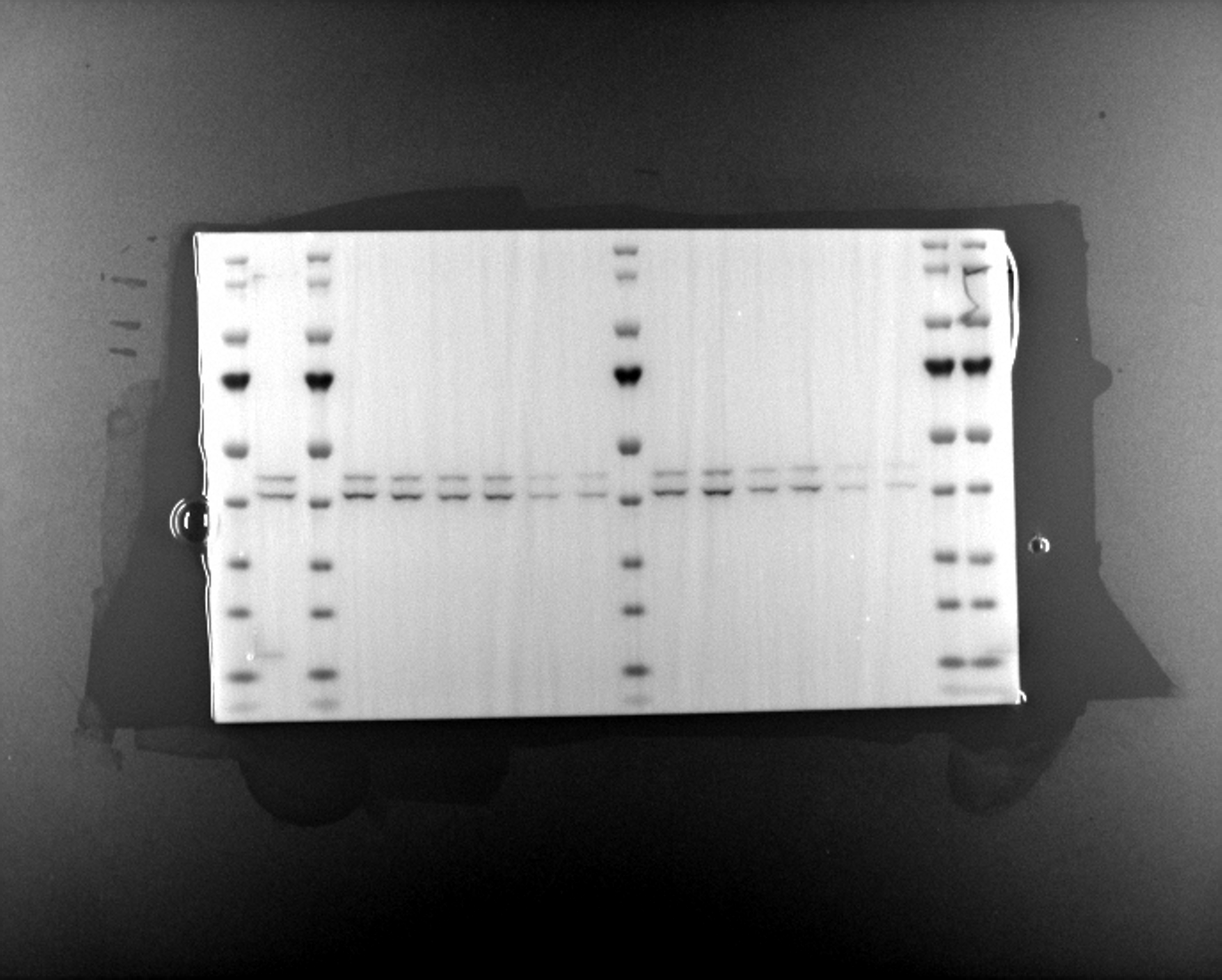
**

**— 50 kDa**

**Supplementary Figure S1b: p-ERK-β-actin**

**
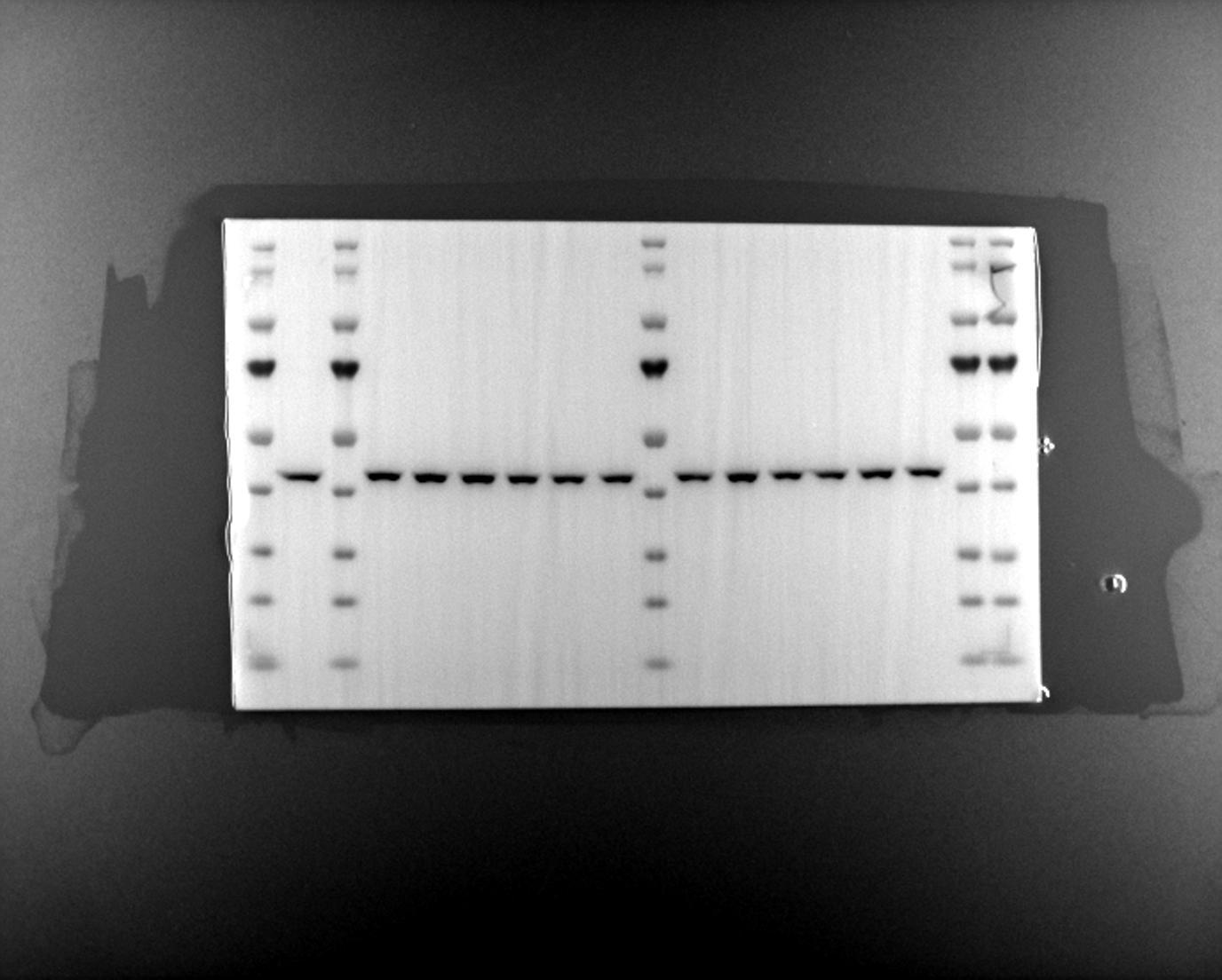
**

**— 50 kDa**

**Supplementary Figure S1b: ERK**

**
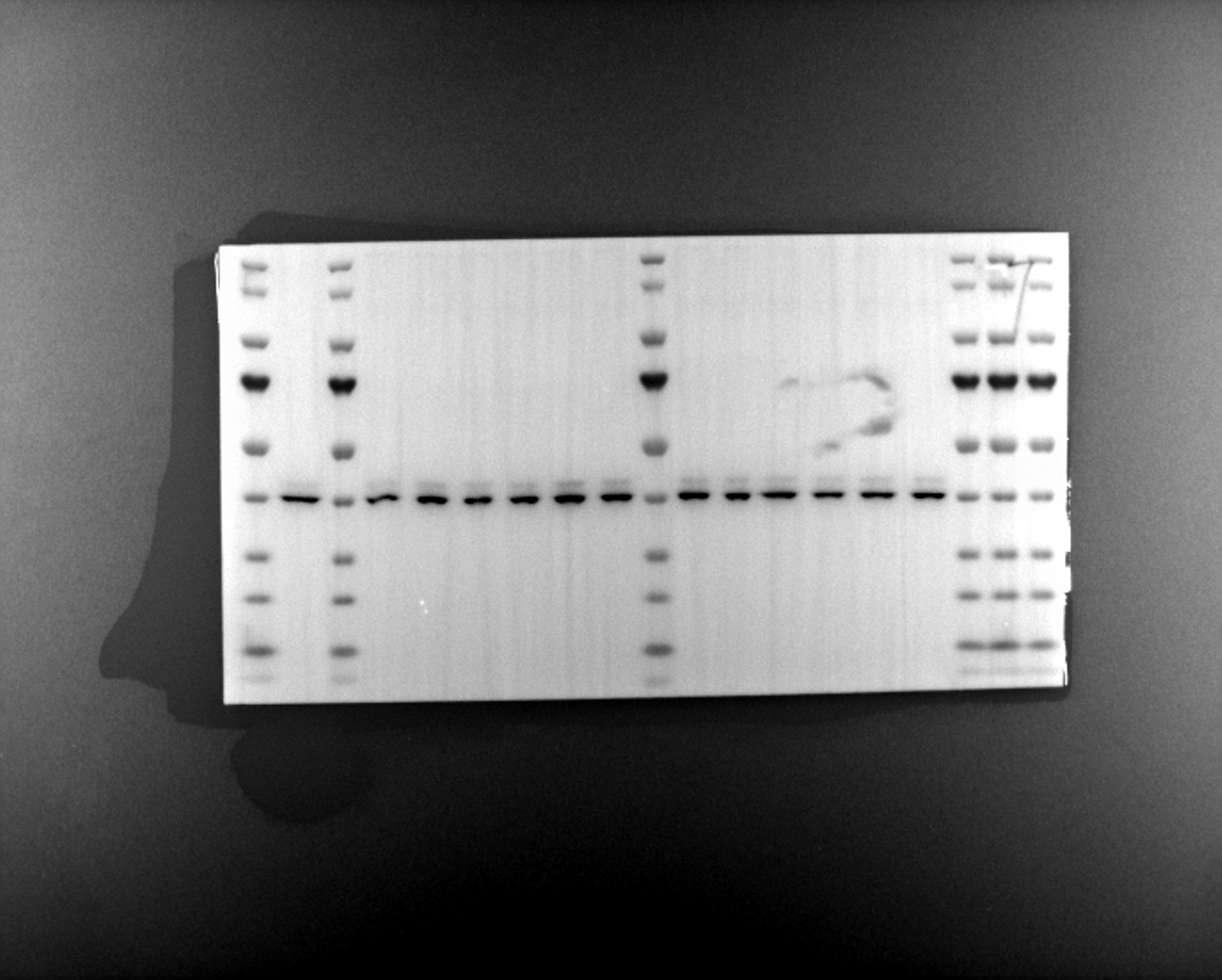
**

**— 50 kDa**

**Supplementary Figure S1b: ERK-β-actin**

**
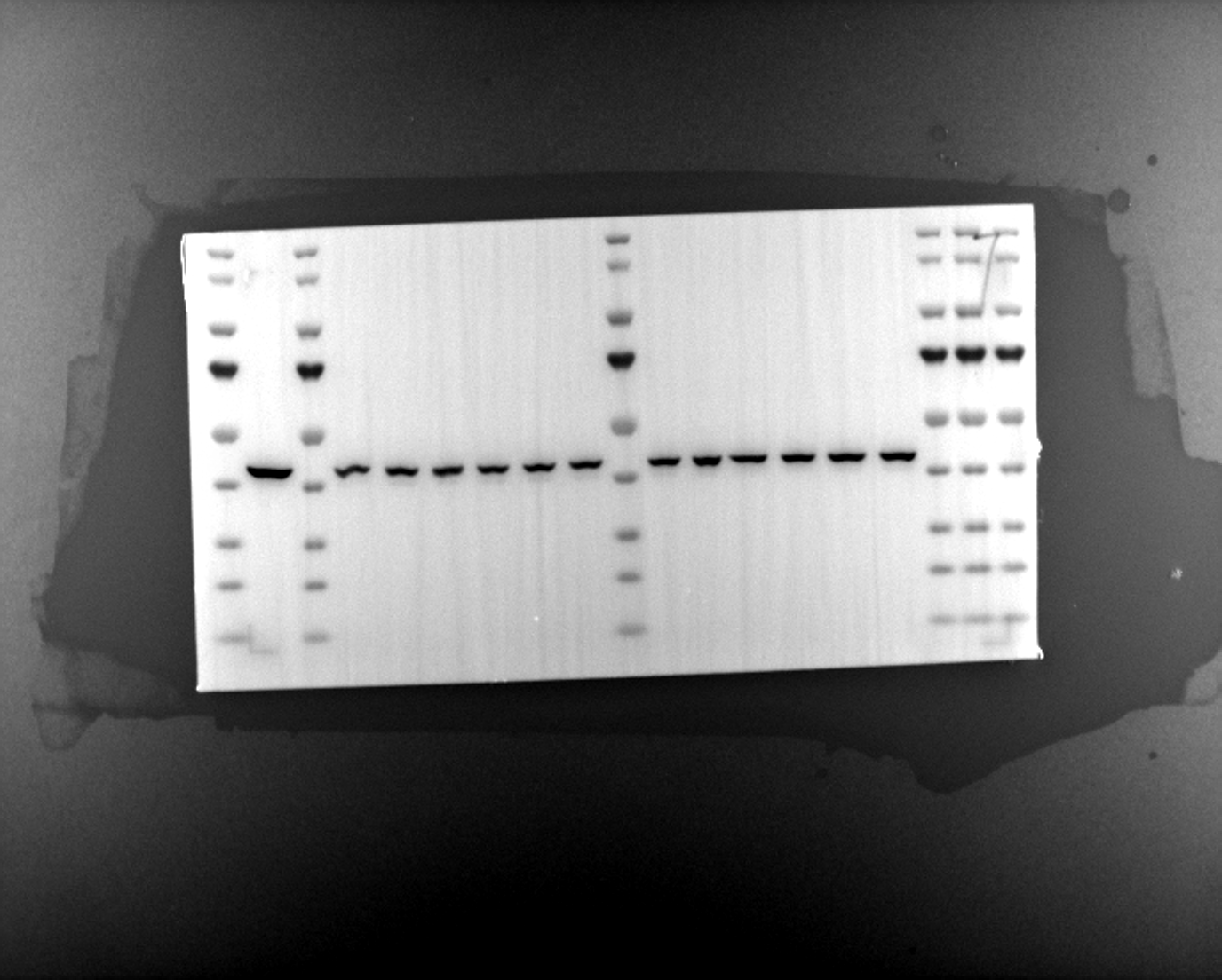
­­**

**— 50 kDa**
